# Dynamics of limited neoplastic growth on *Pongamia pinnata* (L.) (Fabaceae) leaf, induced by *Aceria pongamiae* (Acari: Eriophyidae)

**DOI:** 10.1186/s12870-020-02777-7

**Published:** 2021-01-02

**Authors:** P. P. Anand, N. Ramani

**Affiliations:** Division of Acarology, Department of Zoology, University of Calicut, Malappuram, Kerala 673 635 India

**Keywords:** Herbivore, Histology-FE SEM, Plant-mite interaction, Eriophyid, *Pongamia pinnata*, *Aceria pongamiae*, ATR-FTIR spectrum, Antioxidative potency, EDAX analysis, Nutrient profile

## Abstract

**Background:**

Galls or the neoplastic growth on plants result from a complex type of interaction between the inducers (Acari, Insects, Microbes and Nematodes) and plants. The present study sheds light on the gall inducing habit of a highly host specific eriophyid mite, *Aceria pongamiae,* on the leaves of *Pongamia pinnata* leading to the production of abnormal pouch like outgrowths on the adaxial and abaxial surfaces of the foliage. Each leaf gall is a highly complex, irregular massive structure, and the formation of which often leads to complete destruction of leaves, especially during heavy mite infestation, and thereby adversely affecting the physiology and growth of the host plant.

**Results:**

The study was carried out by making comparative observations on FE-SEM histological sections of galls representing four different growth stages categorized on the basis of difference in age groups. Apart from variations in cell metaplasia, a dramatic change was observed in the abaxial-adaxial polarity of the laminar surfaces also throughout the developmental sequence of galls, in all the four growth stages. Significant variations could be observed in the anti-oxidative potency as well as elemental composition in the all the four age groups of galls, and also revealed ATR-FTIR pattern of gall formation.

**Conclusion:**

Being the first attempt to unravel the mystery of gall induction by eriophyids in general and by *A. pongamiae* in particular, on its host plant *P.pinnata,* by shedding light on the structural and histological alterations taking place during leaf gall formation under the influence of the mite, the current study is to be treated as the model of plant-animal interactive system.

**Supplementary Information:**

The online version contains supplementary material available at 10.1186/s12870-020-02777-7.

## Background

Insect-plant interactions are designated to be either direct or indirect, with a history dating back to the evolution of animals [1]. Plant galls or the cecidia are unique products of interspecific association between plants and organisms, like insects, mites, nematodes, microbes etc. and in a general perspective, resemble abnormal growths. These are the excellent examples to reflect highly host specific co-evolution patterns of phytoparasitic ability to reprogramme the normal development of plant tissues [2] to create comfortable microenvironmental niches within the host plant tissue [3]. These abnormal vegetative tumors develop through the feeding/ovipositional or other mechanical stimuli, exerted by the inducer(s) on the plant tissues to manipulate the tissue programming pattern leading to hyperplasia and hypertrophy [1, 4, 5]. Most of the eriophyid mites are highly host specific, showing extreme preference to feed on the meristematic and young soft tissues of plant organs which grow above the ground level, being highly rich in nutritional resources. This type of astonishing selectivity of these mites very often results in inducing a wide range of symptomless to toxemic effects on their host plants as well as the development of varied symptoms ranging from simple to highly complex types such as russeting, curling, blistering, silvering, bronzing, distortions, necrotic lesions, bud deformations, erineal patches and pouched galls, witches broom effect, stunted growth and so on [5–10].

During the development of galls, a series of anatomical and structural variations takes place, which would affect the plant organs [2]. During their developmental process, galls pass through three major phases viz. the initiation (induction), growth and development, maturation (differentiation) and senescence and the gall design (morphological architectural appearance) is based on the arrangements of tissues [11]. Most of the researchers focused on the cecidogenesis mechanisms and explained the interaction between phyto-parasites and host plants. The pouch/finger like galls represent one of the commonest leaf galls induced by eriophyid mites by injecting saliva into the abaxial epidermis when they suck sap from the plant cells [5, 6]. The free-radical scavenging assay addressed basic questions in gallogenesis, such as why cell damage was observed only in the initial stages and was not observed in senescent stages. The ROS (reactive oxygen species) cascade system is activated while the initial feeding time, it will alter the normal physiological activity of the cells or tissue systems. In classical concepts that, after initial herbivore attacks, the plant immune system is activated and produces several secondary metabolites (especially phenolic compounds), these metabolites play a vital role in inhibiting ROS cascade mechanisms and also reduce the cellular damages [6, 12, 13].

The cecidogenic behavior of a highly host specific, phytoparasitic eriophyid mite, *Aceria pongamiae* which induces pouch/finger like epiphyllous or rarely hypophyllous leaf galls on its host plant, *Pongamia pinnata* (L.) Pierre (Fabaceae) has been illustrated in the present study, as a model system for mechanism of gall formation. So, far, the FE-SEM method has not been applied in the developmental process, and most of the study focused on the characterization of a specialized structure in a tissue system, as well as EDAX or EDA (Energy dispersive X-ray analyzer) X-ray spectroscopic method widely used to analyze the elemental composition of a physical material. In recent years, ATR-FTIR (Attenuated total reflectance- Fourier-transform infrared spectroscopy) spectrum analyzes have been popular in plant taxonomy because they are highly sensitive for functional group characterization of plants under abiotic or biotic stress. In the case of gallogenesis study, shifts in the frequency of absorption bands and changes in relative band intensities indicate changes in chemical structure or changes in the environment around the gall region [14–16]. In this study, we conducted research on ultrastructural histological modification during gall formation using FE-SEM. The selected tools, such as ATR-FTIR and EDAX, are very effective in characterizing minute variation during gall formation. The aim was to identify source-sink nutrient profiling (using EDAX method) and functional group disparities (using ATR-FTIR) in gall formations, while also enlightening the cellular response of the host plant using the DPPH free radical scavenging assay and histochemical profiling. The DPPH assay was used to understand the relationship between cellular necrosis and the degree of free radical scavenging activity of each gall stage. In classical concepts, the free-radical scavenging molecule inhibit the ROS cascade and eventually reduce cellular necrosis. The outcome of each data is used very effectively to define the basic properties of different gall stages and to provide new insights into gall formations.

## Results

**Fig. 1:**
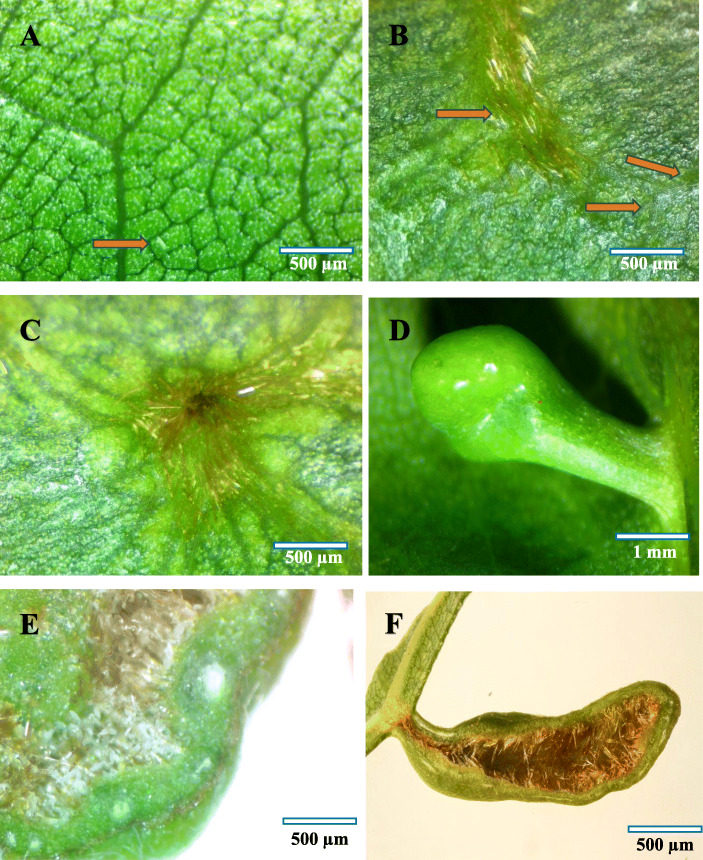
**a** Abaxial surface of normal leaf with the eriophyid mite (red arrow), **b** initial stage of gall formation (within 24 h.) showing the erineal growth (red arrow), **c** Abaxial surface of galled leaf showing the ostiole region of the gall, **d** Abaxial surface showing a mature finger like gall, **e** Cross section of gall showing eriophyid mite population, **f** Cross section of a mature gall showing inner gall cavity with massive growth of hairs

The present research was carried out between late February to July, which was the most favourable time for *A. pongamiae* for induction of gall on its host. The healthy ungalled leaves of the plant were characteristically dark green on the adaxial surface as compared to the abaxial surface. Both the surfaces had a mosaic pattern of green patches and were devoid of trichomes (Fig. 1a).

**Fig. 2:**
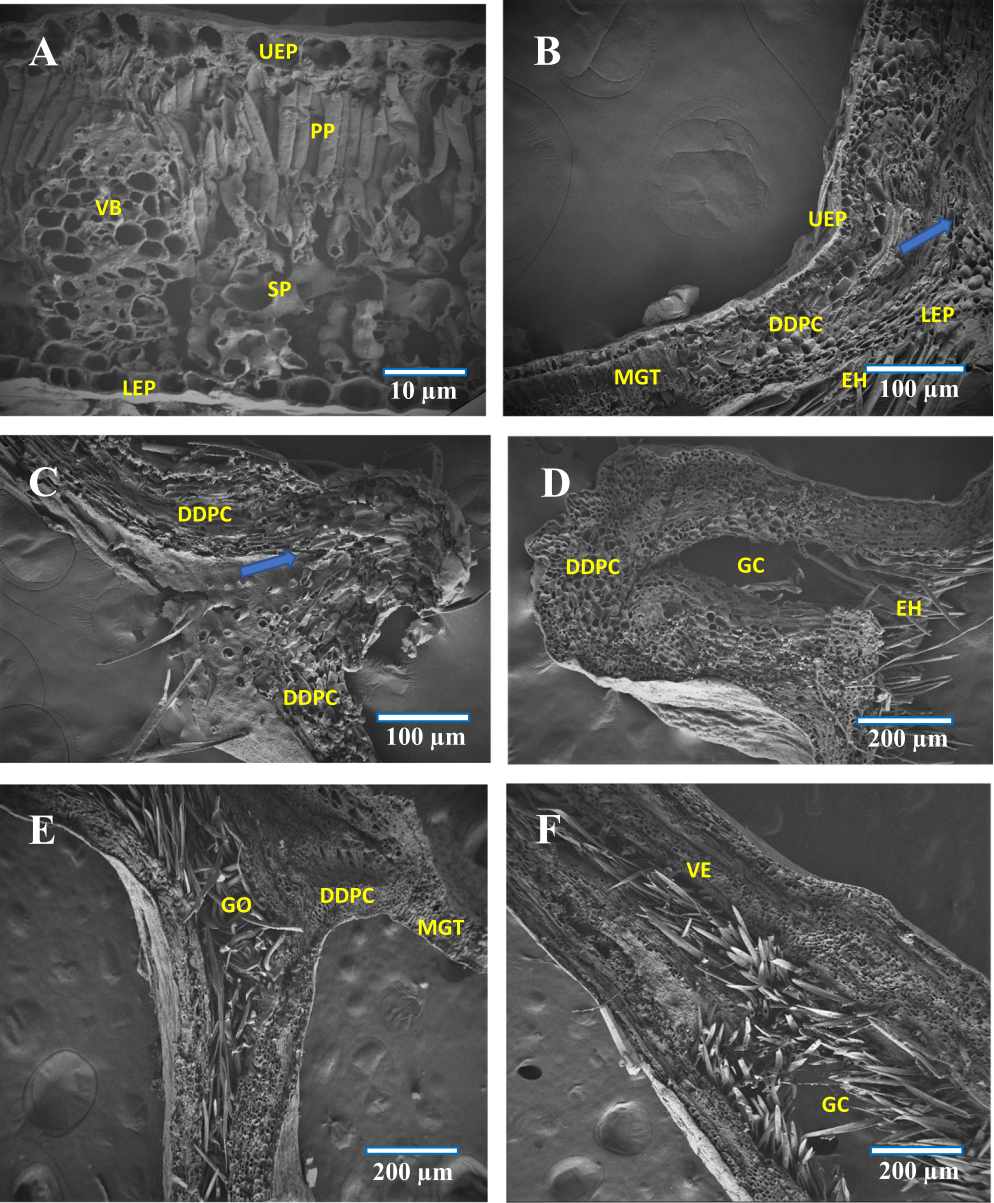
FE-SEM images of (**a**) Abaxial surface of normal leaf showing stomata (arrow), **b** Normal Stomata, **c** Undifferentiated stomata in galled area, **d** Adaxial surface of normal leaf, **e** Upper surface of galled leaf showing longitudinal wrinkled lines, **f** Epiphyllous galls at initial stage of development. S-Stomata, GC- Guard cell, SO-Stomata opening, EE-Epidermal hair

Initiation of mite infestation was identified mainly based on the formation of erinea and distortion of photosynthetic mosaic pattern at the abaxial surface of leaves. Subsequent to mite infestation, the abaxial surface developed several longitudinal wrinkled lines (Fig. 1b), as a result of progressive feeding activity of the mite. These lines were found to originate from different places and joined together at a central axis of pre-determined gall ostiolar region, and which later formed the gall ostiole region (Fig. 1c), during the course of development of the gall. Two days after gall induction by the mite, the adaxial leaf surface developed small tubercle like structures. Initially, the upper surface of galls appeared smooth, and a transition stage was recognized between the first (1–3 day) and second stage (10–13 days) of development of the gall. A thick waxy cuticular covering was developed at the transition stage/zone and up on which, large and stout trichome hairs were found to develop (Fig. 2f). The erineal hairs were transparent and light greenish in color at the initial stage of development as the chloroplast got shifted to the erineal hairs from the lower epidermis. With progressive aging of the gall, the amount of chloroplast got reduced in the erineal hairs and the latter assumed a reddish-brown tinge owing to the deposition of coloured pigments (Fig. 1f). A direct correlation could be recorded between the age of the gall and the structural complexity, both morphologically and histologically.

During the early stage of gall development, the mites were found to exhibit a unidirectional mode of feeding, by concentrating at the inner terminal region of the gall, imparting a finger like appearance to the gall. During the second stage of development (after 10 days of gall development), the mite population density showed a progressive increase (Fig. 1e), displaying diverse feeding trends in an irregular direction, thereby culminating in varied forms of alterations in the internal tissue organization of the gall. Accordingly, the gall morphology also was changed from the finger like (Fig. 1d) form to highly complex and often irregular structures (Fig. 1f).

### Control leaf

**Fig. 3:**
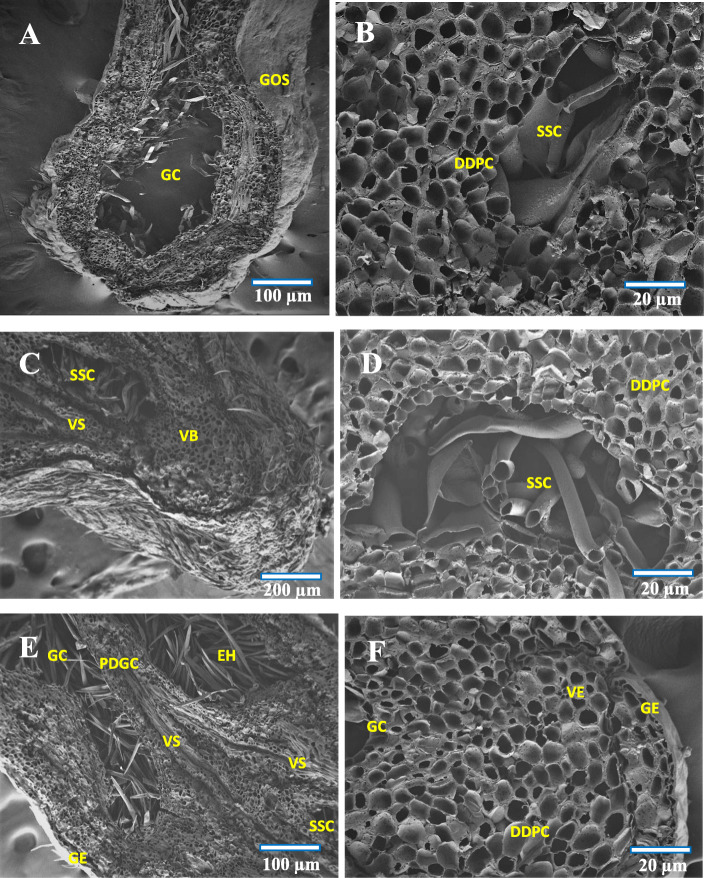
FE-SEM cross section images of (**a**) Normal leaf, **b** Initiation of gall formation - dedifferentiation of well differentiated tissues, **c** Gall cavity formation initiated, **d** 2nd stage, uncontrolled cell division, **e** 3rd stage, limited cell division at gall pedicel region, **f** 3rd stage – gall cavity enlargement. VB-Vascular bundle, SP- Spongy tissue, PP-Palisade tissue, UEP-Upper epidermis, LEP-Lower epidermis, MGT-Mesophyll ground tissue, DDPC-Dedifferentiated parenchyma cells, EH-Erineal hairs, GC-Gall cavity, GO-Gall opening, VE- vascular epidermis

In the ungalled leaf, a single layer of compactly arranged epidermal cells was visible both on the adaxial and abaxial surfaces. The abaxial epidermis was found to possess paracytic type of stomata (Fig. 2b) and was devoid of trichomes. The mesophyll was found to comprise ground tissue (palisade and spongy parenchyma). The palisade parenchymatous tissue was found to comprise two layers of compactly arranged columnar cells and the spongy parenchymatous tissue was formed of loosely arranged 4–5 layers of large round and oval shaped cells with small intercellular space. The spongy tissue contained comparatively lesser number of chloroplasts than that of the palisade tissue (Fig. 3a).

### Histology

Within 40–45 days, the gall attained complete maturity, and after the maturation phase, no further histological organization was observed, and hence this gall was categorized as limited neoplastic growth. In response to the mechanical and chemical stimuli perceived from the inducer, the lower epidermal cells initiated the triggering mechanism for gall formation. Subsequently, the well differentiated mesophyll tissue was found transformed into undifferentiated meristematic tissue. In the first stage of development (1–3 days old), the mesophyll tissue got dedifferentiated into actively dividing meristematic tissue (Fig. 3b).

The mesophyll tissue of the abaxial surface became hyperplastic and underwent repeated anticlinal divisions before getting hypertrophied. The highest rates of mitotic divisions were evident, with formation of uniformly distributed mass of parenchyma cells in different shape and size. As a result of the numerous anticlinal and periclinal divisions which occurred at the parenchymatous tissue, an initial uplifting of tissues occurred on the adaxial leaf surface, which assumed the form of a small tubercle (the gall being an actual extension of upper epidermal cells). Due to the uncontrolled cell multiplication occurred at the abaxial surface, the chloroplast got reduced and simultaneously, an upward pushing of abaxial epidermis was observed (Fig. 3b-c). Formation of gall cavity was initiated through cell necrosis which occurred at the hypersensitive region (Fig. 3d). Subsequent to this, an increase in the growth of uniseriated erineal hairs was observed, thereby covering the abaxial epidermis.

**Fig. 4:**
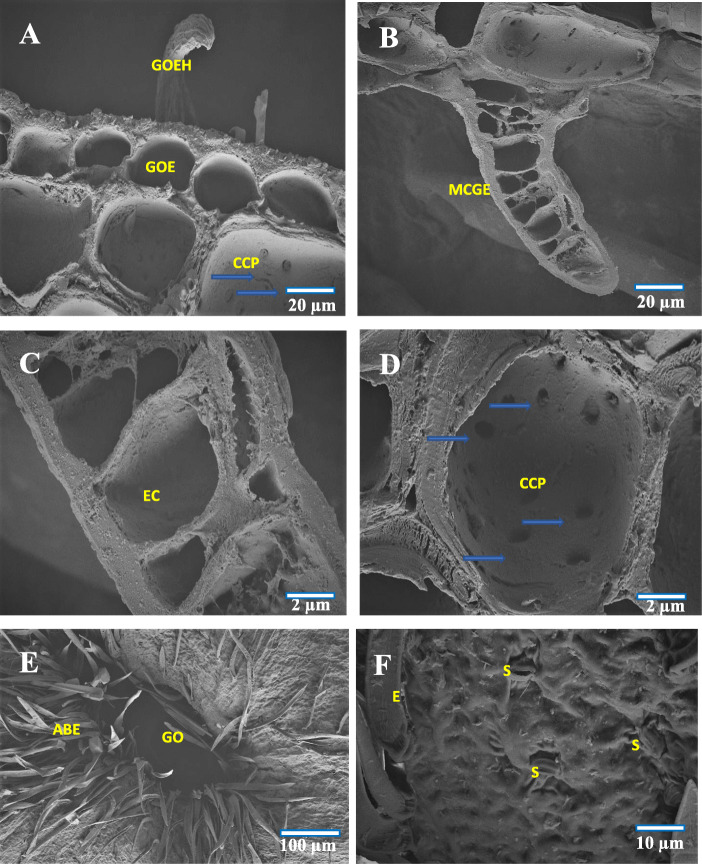
FE-SEM cross section images of (**a**) Enlarged view of gall epidermis, **b** Multicellular erineum, **c** Enlarged view of multicellular erineum, **d** Enlarged view of cells with intercellular connection pit (blue arrow), **e** Abaxial surface showing gall ostiolar opening with erinea, **f** Abaxial surface of gall with stomata and erinea. GOE-Gall outer epidermis, GOEP-Gall outer epidermal hair, CCP-Cell with connection pit, GO-Gall opening, ABE-Abaxial erinea, MCGE- Multicellular gall erinea, E- Erinea, S-Stomata, EC-Erineal cell

In the 2^nd^ stage of development (about 10–13 days old galls) which represented the gall primordium stage, the actual growth and development of the gall started. At the lower part of the gall, the cells became continuously proliferated, and the length of gall got increased through periclinal cell divisions and thickening of gall occurred through anticlinal cell division of dedifferentiated parenchyma cells. The vascular elements were developed from the compactly arranged meristematic parenchyma. These vascular elements were arranged as broken rings and were encircled with compactly arranged parenchyma cells (Fig. 3e). Formation of multicellular erineal hairs occurred from the inner layer of compactly arranged parenchyma cells, and which protruded into the gall cavity (Fig. 4b).

**Fig. 5:**
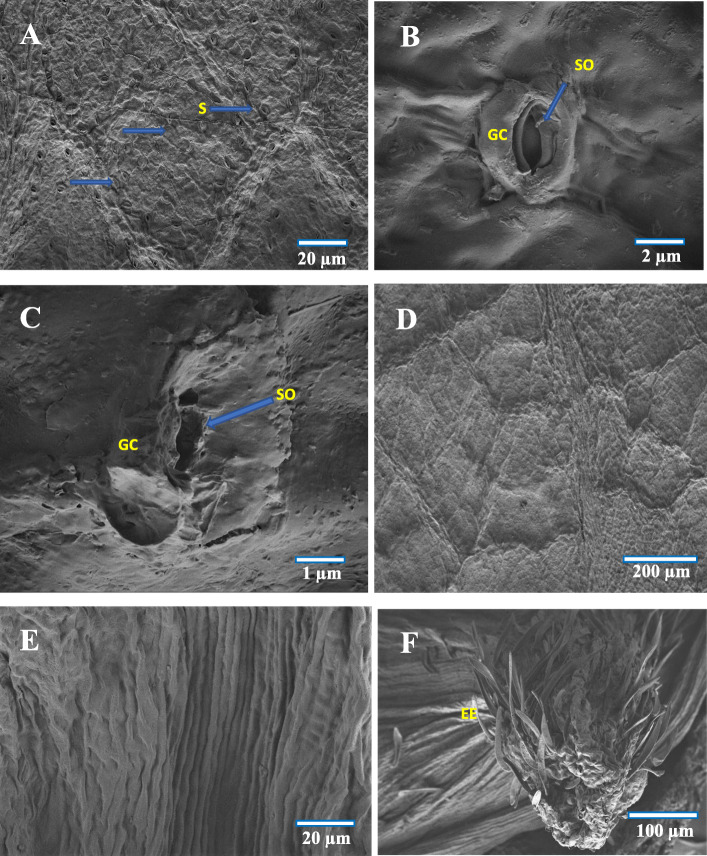
FE-SEM cross section images of (**a**) Gall in mature stage of development, **b** Schizogenous secretory cavity formation initiated, **c** Developed schizogenous secretory cavity, **d** Enlarged view of schizogenous cavity, **e** Initiation of gall cavity separation, **f** Enlarged view of matured gall tissue. GC-Gall cavity, GOS-Gall outer surface, DDPC-Dedifferentiated parenchyma cells, SSC-Schizogenous secretory cavity, VS-Vascular strands, VB-Vascular bundle, PDGC- Partition of gall cavity, GE-Gall epidermis, VE-Vascular elements, EH-Erineal hairs

At the opening region of the gall (ostiole), the erineal hairs were large, thick and densely arranged (Fig. 3e) while those at the inner gall chamber were thin and loosely arranged (Fig. 3f). At this stage, the gall was found to possess a basal stalk (pedicel) attached to the leaf adaxial surface (epiphyllous condition) and an upper thick cell mass of globular structure (Fig. 1d). The gall chamber was formed through necrosis and programmed cell death and which could lead to formation of young pouch like/finger like gall. Formation of vascular strands and sclerenchyma cells occurred by redifferentiation of proliferated parenchyma cells. The cells of the lower epidermis and sub-epidermis were found transformed into interior tissue of gall cavity with less chloroplast content. Around the gall chamber, 5–7 layers of parenchymatous cells were observed, serving the function as nutritive tissue. During this stage, initiation of schizogenous secretory cavity formation also became evident (Fig. 5b).

During the maturation phase (3^rd^ stage, about 25–27 days old gall), the gall primordial activity was reduced. The cells at the lower region of the gall were found continuously proliferated through periclinal division and the differently sized and structured new daughter cells pushed the nearby cells in to the outer epidermis of the gall. During this stage, the periclinal division at the basal and pedicel regions of the gall was reduced and the anticlinal division increased. Alterations in this type of cell division, would affect the gall opening (narrow ostiolar opening) and gall cavity formation (voluminous internal cavity). Formation of vascular strands and vascular elements was found increased, and about 10–15 vascular elements were arranged in the homogenous meristematic parenchyma cells. The proliferated parenchyma cells were found redifferentiated into sclerenchyma cells of different size and distributed infrequently in the outermost layer of dividing parenchyma cells (Fig. 5a and f). With the formation of a schizogenous secretory cavity in the dedifferentiated parenchyma region and the anticlinal and periclinal division of the terminal parenchyma cells, the gall morphology got altered. In this stage, not much histological changes were observed, though a few cells underwent limited periclinal division. The major changes at this stage of gall development were the thickening of the erineal hairs at the ostiolar region (Fig. 4e) and rarely the secondary outgrowths which developed for separating the gall cavity into chambers (Fig. 5e). The striking feature at this stage was the thickening of vascular strands and elements, and the reduction in chlorophyll of the outer epidermal cells of the gall.

The 4^th^ stage, which constituted the initiation of senescent stage (about 40–43 days old stage), in this stage histogenesis was completely stopped. In some rare conditions, limited secondary interior outgrowth of cells occurred through periclinal cell divisions. However, formation of thick vascular elements was evident through limited anticlinal division. The compact arrangement of parenchyma cells was found reduced in between the gall interior endodermis and the outer epidermis along with the formation of more than one schizogenous secretory cavity and thickening of outer and inner gall tissues. The polarity behind the adaxial (upper) and abaxial (lower) surfaces of the leaf got completely changed at this stage, and programmed cell death and necrosis also started. Progressively, the gall became dead and dried off (Fig. 1f).

### Histochemical profile

**Fig. 6:**
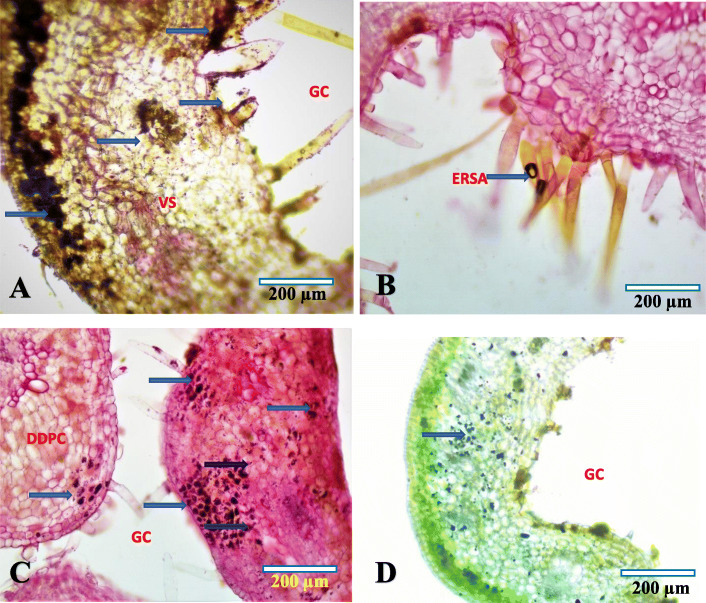
Histochemical analysis of matured gall based on light microscopic images (**a**) Phenolic accumulation (arrow), **b** Reducing sugar in erinea (arrow), **c** Starch accumulation in the nutritive zone of gall (arrow), **d** Protein accumulation. GC-Gall cavity, VS-Vascular strand, ERSA- Erinea with reducing sugar accumulation, DDPC-Dedifferentiated parenchyma cells

Gall tissue gradients and source-sink alterations were studied based on the various primary and secondary metabolites. The primary metabolites studied were the reducing sugar, starch and protein while the secondary metabolite studied was the phenolic compounds. Accumulation of phenolic compounds could be noticed from the 2^nd^ phase of gall development onwards, and the major site of phenolics accumulation was identified mainly in the outer 2–3 layers of the hypodermis region. Phenolic compounds were found irregularly distributed in the inner 1–2 layers of the gall nutritive zone. Galls in the initial maturation phase (25–27 days old galls) showed high level of phenolic accumulation in the outer and inner parenchyma tissue regions. The 4–6 layers of hypodermis showed the highest phenolic accumulation when compared to the inner tissue zone. Irregular patch like phenolic distribution was observed in 2–3 layers of the nutritive zone. Phenolic accumulation could be observed in the endodermis region of the schizogenesis secretory cavity and also along the lateral side of multicellular erineal hairs (Fig. 6a). Primary metabolite gradients also could be traced in the maturation phase of gall tissues. Presence of starch granules was evident in the mesophyll regions and inner gall tissue layers. The major sites of starch accumulation observed during the study were the 2–6 layers of hypodermis region and the 2–3 layers of inner nutritive zone. Starch was found absent in the actively dividing dedifferentiated parenchyma cells. Deposition of amyloplasts was noticed in the vascular elements and in the surrounded parenchyma cells. Uniform distribution of starch was observed at the basal region of erineal hairs (Fig. 6c). Presence of reducing sugar was observed mainly in the actively dividing tissues of parenchyma cells. Very low amount of reducing sugars could be noted in the outer and inner compactly arranged cellular regions. In the 2^nd^ stage of gall development, the reducing sugar was found accumulated in the 4–6 layers of hypodermis and inner nutritive zones. During the transition period from the 2nd stage to the growth phase (25–27 days old), the middle-dedifferentiated parenchyma cells underwent several mitotic divisions, and the reducing sugars moved from the hypodermis and nutritive zone to highly active proliferated regions. The major site of reducing sugar accumulation was observed to be the multicellular erineal hairs inside the gall (Fig. 6b). Protein accumulation was observed in the middle-aged gall (25–27 days old gall) in which the protein was found precipitated and accumulated throughout the gall tissues. High level of protein precipitation was recorded in the middle zone followed by inner nutritive zone (Fig. 6d).

### Antioxidative potency

**Fig. 7:**
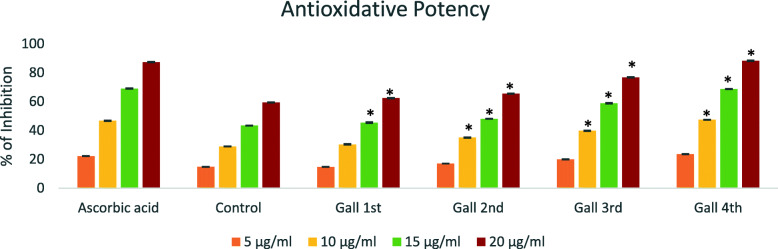
Graphical representation of Antioxidative potency of different developmental stages of gall with control leaf and Positive control (Mean ± SD). The percentage of inhibition indicated as the, this much amounts of plant extracts (5–20 μg/ml) needed for scavenging or inhibiting the fixed concentration of DPPH free-radicals. (* Indicates significance level at 0.05)

In DPPH scavenging assay, the Vitamin C (Ascorbic acid) was used as the standard (positive control) and its IC_50_ value being 11.05 μg/ml. The methanolic extracts of the control (uninfested) leaves of *P.pinnata* and its galls in different developmental stages showed significant variation to neutralize the DPPH free radical. The antioxidant potential of the control leaf was 16.69 μg/ml and the different developmental stage of galls showed a range of antioxidant capacities. The IC_50_ value of the gall in the initial stage (1–3 days old galls) of development was 15.45 μg/ml and subsequent IC_50_ values were 15.21 μg/ml, 12.81 μg/ml and 10.89 μg/ml respectively for the 2^nd^ stage (10–13 days old galls), 3^rd^ stage (25–27 days old galls) and 4^th^ stage (40–43 days old gall) (Fig. 7).

However, the antioxidant activities were lower than that of the positive control ascorbic acid (IC_50_ was 11.05 μg/ml). Antioxidant capacity of the methanolic extract of the control leaf as well as the galls in different stages of development showed significant variations (*p* < 0.05). The values for the antioxidant capacity of the gall tissues were comparatively higher than those of the control leaf tissue and the values showed an increase with respect to the increasing age of the gall. Thus, a direct correlation could be established between the age of the gall and its antioxidant capacity. However, the antioxidant capacity of the galls in the first and second stages of development showed only slight variation.

### FE SEM-EDAX analysis

Results of the EDAX analysis (Supplementary file: Table 3) showed highest concentration of Carbon (43.34 ± 1.64%) at the non-vascular bundle region of the control leaf when compared to that of the vascular bundle region (41.86 ± 0.02%). The mean elemental concentration at the vascular bundle region of the control leaf could be depicted as: Oxygen (26.04 ± 0.03%), Nitrogen (1.05 ± 0.04%), Phosphorous (8.55 ± 0.03%), Potassium (19.42 ± 0.03%) and Calcium (0.046 ± 0.010%).Whereas the concentration of the above-mentioned macronutrients in the tissue region of the control leaf could be recorded as: Oxygen (27.24 ± 0.035%), Nitrogen (0.46 ± 0.02%), Phosphorous (7.53 ± 0.03%), Potassium (15.94 ± 0.02%), Calcium (0.34 ± 0.02%) and Magnesium (0.22 ± 0.016%). The major micronutrients considered for the analysis were Iron, Chlorine, Manganese, Zinc, Copper, Boron, Molybdenum and Cobalt. In the control leaf, the non-vascular bundle region showed the absence of Iron and Cobalt while the vascular bundle region showed the absence of four nutrients viz. Iron, Cobalt, Copper and Zinc.

**Fig. 8:**
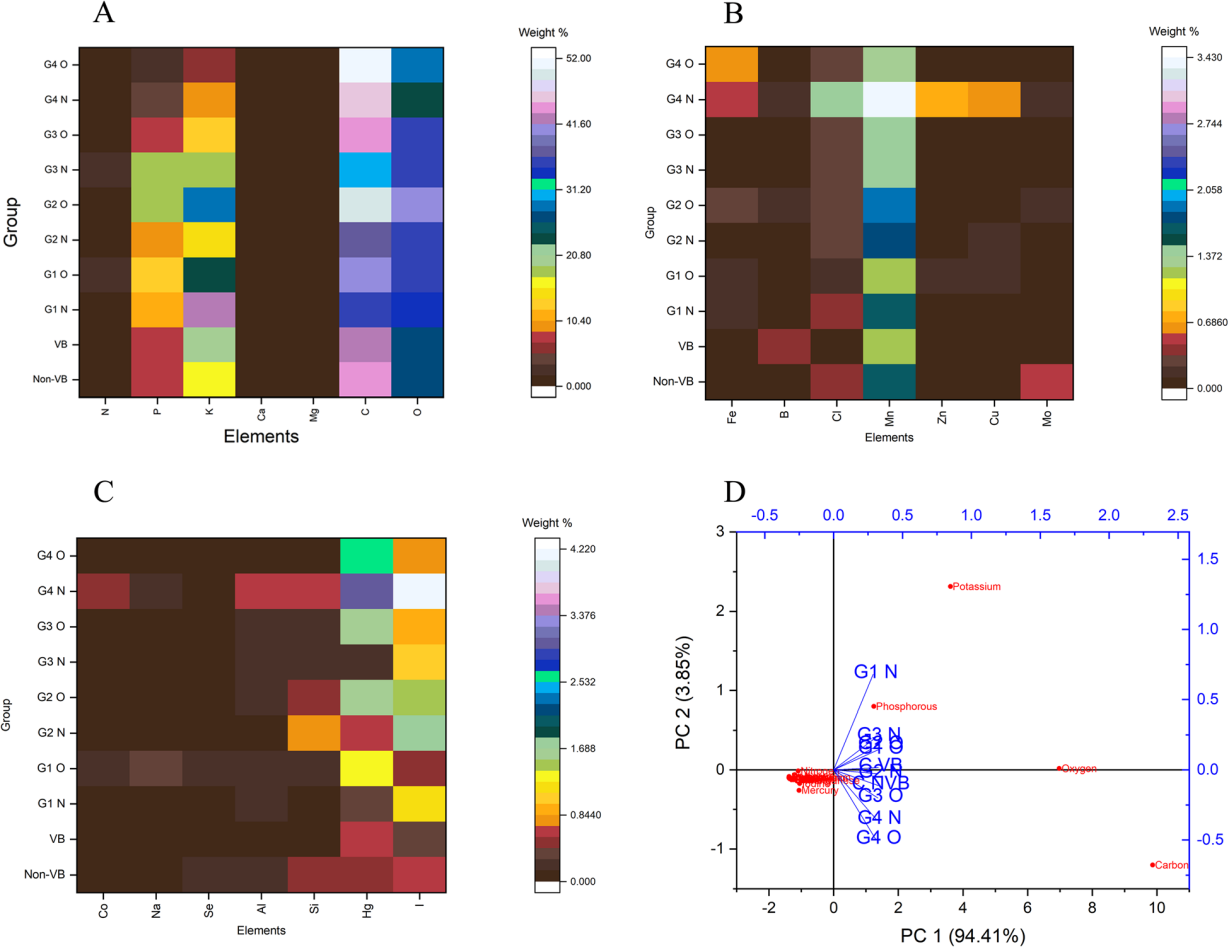
source-sink elemental analysis of different developmental stages of galls with comparing the inner and outer region of galls (**a**) macronutrients (**b**) micronutrients (**c**) other elements (**d**) PCA-Biplot showing the relationship between each group. CVB: Control vascular bundle, C NVB: Control non-vascular bundle, G1 O: Gall 1^st^ stage outer region, G1 N: Gall 1^st^ stage inner region, G2 O: Gall 2^nd^ stage outer region, G2 N: Gall 2^nd^ stage inner region, G3 O: Gall 3^rd^ stage outer region, G3 N: Gall 3^rd^ stage inner region, G4 O: Gall 4^th^ stage outer region, G4 N: Gall 4^th^ stage inner region

The percentage of macro (Fig. 8a) and micro (Fig. 8b) nutrients present in the inner and outer regions of the galls in different stages of development was also analyzed to understand their source-sink status. Higher concentration of most of the elements was observed in the inner region of the gall. Concentration of Aluminum, Silicon and Selenium showed variation with respect to changes in the stage of development of galls (Fig. 8c). Concentration of mobile nutrients like Nitrogen showed direct correlation with the age of the gall as evidenced through the results of the study. The concentration of nitrogen in the inner region of the galls was initially low and it increased with the age of the gall up to the 3^rd ^stage of development. After this stage, all the nutrients in the inner region of the gall showed a decrease. Accumulation of Mercury (Hg) was also observed in the leaf, typically Hg is highly toxic to herbivores and plants. In the study, the concentration was 0.50 ± 0.13 at the non- vascular bundle region of the control leaf and 0.67 ± 0.02 at the vascular bundle region. Initially, the outer region of the gall showed high level of mercury concentration and with the advancement of gall development, mercury level decreased in the outer region while that of the inner region showed an increase, reaching 2.98 ± 0.023% in the inner region of the 4th stage of gall. The percentage of Iodine was significantly higher at the inner region of the 4th stage gall (4.12 ± 0.02%) when compared to that of the control leaf at the Non-Vascular bundle region (0.67 ± 0.015%).

Results of FE SEM-EDAX elemental analysis of various developmental stages of leaf galls helped to understand the elemental distribution pattern (Supplementary File: Table 3). Results of PCA of control leaf against the four different developmental stages of the gall showed significant variation in elemental distribution. The correlation analysis (Supplementary File: Table 2) of galls in different stages against the control leaf showed significant variations. PCA biplot (Fig. 8d) showed large positive co-variance, showing linear relationships between other variables. Based on eigenvalues and Scree plot, total two PCA covered 98.26% variance. The biplot explained how much correlation present between each gall developmental stage. Most of the vectors (different stages of gall) showed high positive correlation, because the vectors were placed close, forming small angle between them except in the inner region of the 1st stage gall (G1 N). Most of the vectors were found highly influenced by PC1. From the analysis, it can be clearly confirmed that the gall acts as a sink, especially the inner gall regions (during gallogenesis – based on correlation of PCA vector angle and correlation matrix, the low angle between the groups indicated as the closely related groups, the angle is increasing between the groups – indicated as the dissimilarities between groups), most of the essential or mobile nutrients for cellular growth and the nutrients for mites (herbivores) have been reached after the initial gall stage. Then also, in the senescent period, the concentration of all mobile and essential nutrients is reduced.

### Attenuated Total reflectance (ATR) analysis

**Fig. 9:**
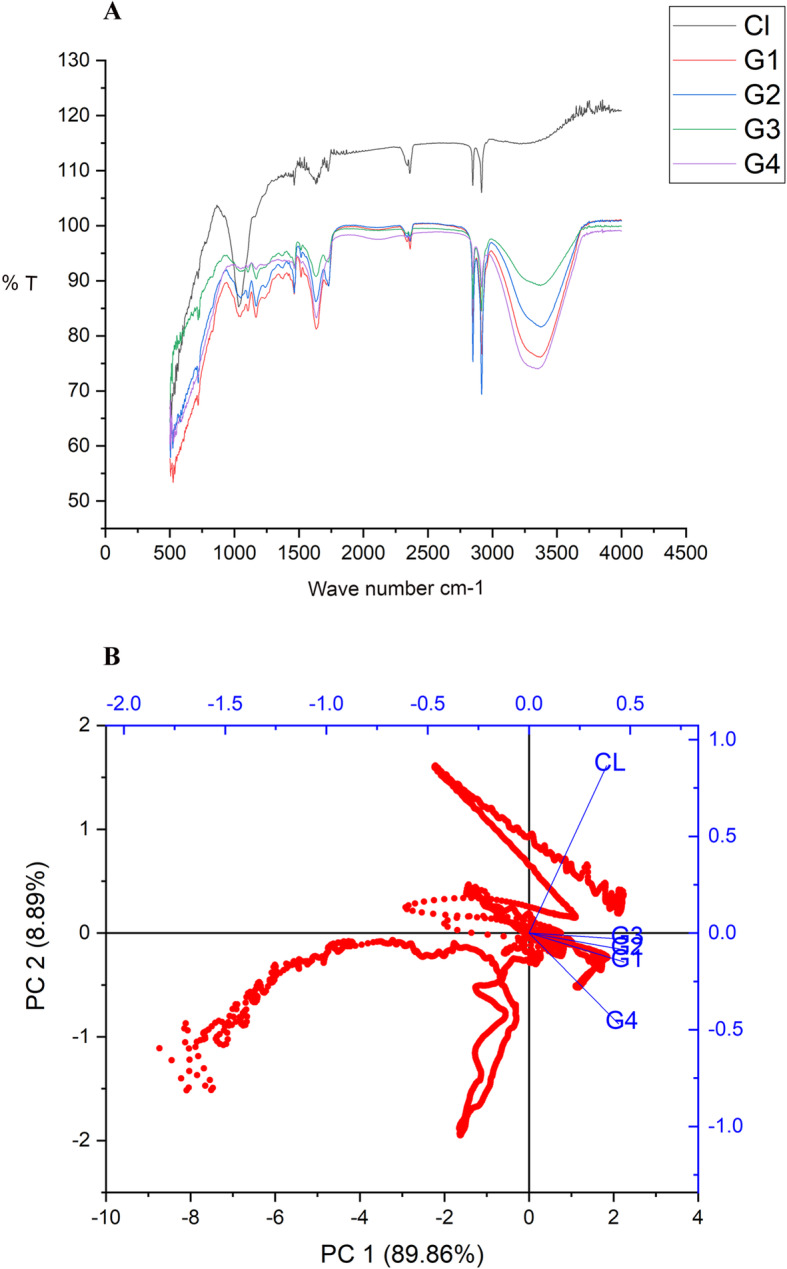
**a** Combined ATR-FTIR spectrum of different developmental stages of the gall and control leaf. Control leaf -Cl, Gall 1st stage – G1, Gall 2^nd^ stage – G2, Gall 3^rd^ stage – G3, Gall 4^th^ stage – G4. **b** PCA Biplot of ATR-FTIR spectrum of different developmental stages of leaf gall. Cl-Control leaf, G1-Gall 1^st ^stage, G2- Gall 2^nd^ stage, G3- Gall 3^rd^ stage & G4- Gall 4^th^ stage

In the control leaf**,** 34 major functional groups were analyzed, while 15 functional groups were recorded in the gall of first stage development, (1–3 days old gall), 13 functional groups in the second stage (10–13 days old gall), 46 functional groups in the 3rd stage (25–27 days old gall) and 16 functional groups in the gall of 4th stage (40–43 days old gall). Thus, the highest number of functional groups could be analyzed in the 3rd stage of development of the gall (Fig. 9a) (Supplementary file: Spectrum 1, 2, 3, 4, 5 and 6, Table 1 & 4).

Chemical class analysis enabled to record the presence of 13-chemical classes in the control leaf, 10 chemical classes in the 1^st^ stage gall, nine chemical classes in the 2^nd^ stage, 23 chemical classes in the 3rd stage and 11 chemical classes in the 4th stage of the gall. The carboxylic acid, alcohol groups, CF3 groups, aromatic and aliphatic functional groups were observed in all stages. The functional variation could also be related to their chemical components present in the gall areas. The absorption peaks at the range of 2849 and 2916 cm^− 1^were observed in all stages of the gall and control leaf. The peaks indicated the presence of CH_3_, CH_2_ functional group vibration. Identification of these compounds could be effectively used as chemical markers to analyze the molecular changes observed in different developmental stages of the gall. Tissue around the 3^rd^ stage was used for spectral analysis for capturing the reason for highest functional groups present in the 3^rd^ stage gall. Tissue around the gall region revealed the presence of 48 functional groups. Correlation matrix analysis of different developmental stages of the gall against the control leaf revealed a positive correlation between the control and the various developmental stages with the values for the 1^st^ gall stage (0.75002), 2^nd^ stage gall (0.76637), 3^rd^ stage gall (0.78806) and 4^th^ stage gall (0.5923) (Supplementary file: Table 4). As shown in the table, the 4th stage gall showed the highest level of variation when compared to that of the other developmental stages of the gall. The spectra of both the mite infested and uninfested leaf samples contained two marker absorption peaks in the range of 2900–2910 cm^− 1^ and 2840–2850 cm^− 1^.

The functional groups were found distinctly distributed under 20 chemical classes, and these chemical classes showed distinct variation in functional group distributions. Seven functional groups were observed under the aliphatic nitrile or multiple bonded nitrogen, six functional groups in alkene, four functional groups in aliphatic carboxylic acid, three functional groups in metal carbonyl and side chain or substituent group chemical classes. Under the chemical classes of aliphatic anhydride, aliphatic silicon compound and aliphatic sulfur compound contained two functional groups and one functional group each was observed under the aromatic amino acid, aliphatic amine, aliphatic amino acid, aliphatic carboxylate, aromatic or conjugated ketone, aliphatic amide, aliphatic ether, aromatic or conjugated carboxylic acid, organic halogen compound, aliphatic thiocompound, aliphatic sulfur compound and aliphatic halogen compound.

Results of PCA of the control leaf against the four developmental stages of the gall showed significant variations in the functional group distribution. The correlation analysis of different stages against control leaf also showed significant variations. PCA biplot (Fig. 9b) showed large positive co-variance, showing linear relationships between other variables. Based on eigenvalues and Scree plot, a total of two PCA was selected which covered 98.93% variance. The chemical properties of the lower epidermis are completely altered during gall formation and the expression of the functional group in the control leaf is completely different from that of the gall. Due to the high degree of biotic stress, the expression of normal functional group is totally altered and stress functional groups are expressed. In the first three stages, there were only slight changes in the functional groups and in the last phase (4^th^ stage) there was a large difference from the control, as can be seen in the PCA plot (Fig. 9b). The spectral functional group analysis supports the hypothesis that biotic stress completely alters the distribution and expression of the molecular structure and functions of functional groups.

## Discussion

Galls, like other regular plant organs, have their own characteristic features like histology and physiology [5, 17]. A high degree of specificity is maintained between the host and the inducer [18], and hence the gall morphogenesis is highly conserved and thus would be useful in the study of gall lineages and cell fate [1]. The actual nature of first stimulus of gall induction in eriophyid mites is currently unknown, but the cell proliferation has been evaluated by several authors in different ways [12, 13, 19]. Results of hormonal and metabolite analysis have clearly revealed the presence of phenolic accumulation in gall tissues during the initial stage of gall development [20]. The phenol rich cells would inhibit/block the action of IAA oxidases and thereby leading to increase in local concentration of auxin. The IAA accumulation would result in cell hypertrophy. The initial stimulation from the inducer would initiate the reactive oxygen species (ROS) cascade events and the ROS signaling leads to phenolic accumulation and sequential molecular events, which are believed to induce gall morphogenesis [21].

Unlike the earlier studies which were mainly focused on the anatomical analysis of mature middle-aged galls alone, in the present study, attention was focused to analyze the different developmental stages of galls. It was observed that the galls completed development within 40–45 days, and in the present study, the selection and categorization of different developmental stages of gall were done based on variations in morphology, anatomy and also on the erineal color pattern. In the initial developmental stage of the gall (1–3 days old), the abaxial epidermis received chemical messengers from the mite saliva and these chemical messengers distorted the normal histological organization of the leaf. The chemical messengers acted on the ground mesophyll tissues, and subsequently well differentiated palisade and spongy parenchyma underwent redifferentiation, leading to uncontrolled proliferation of dedifferentiated parenchyma cells [12, 20–22]. Initially, the hypertrophy and hyperplasia conditions were observed. These findings were in agreement with the previously recorded findings on the leaf gall induced by *Fragariocoptes setiger* on *Fragaria viridis* [5]. Results of histochemical studies enabled to record highly active histological reorganization during the growth phase (25–27-day old galls). The major gall tissue was found to consist of proliferated dedifferentiated parenchymatous cells [21]. The hypertrophied and hyperplasic tissues were found to contain several nutrients and metabolites. Abnormal cellular organization could be noticed without any perfect tissue organization [5, 21].

Results of histological reprogramming assessment of leaf galls carried out during the present study clearly revealed the anatomical and morphological characterization of different developmental stages of galls and also the patterns of gall organogenesis. The morphogenesis pattern of gall development presented various levels of manipulation induced by *A.pongamiae*, indicating that the gall actually represents a limited neoplastic growth. Anatomical characterization of galled and ungalled leaves revealed distinct variations from that of the control leaves [7, 21, 23]. The galled leaves did not show any differentiation between ground tissues, and only a homogenous mass of dedifferentiated mesophyll tissues could be observed in the galled leaves. But, a perfect distribution pattern of ground tissues and vascular bundles was observed in the non-galled leaves. The anatomical alterations induced initially by *A.pongamiae* were well studied [24]. The salivary phytotoxins secreted by eriophyid mites while sucking up the cell sap induces anatomical and morphological differences, leading to the gall formation [6, 8, 10, 11, 25].

In the present study, the antioxidant capacity of the galls in different developmental stages was evaluated and compared with that of control leaves. In the initial stage (1–3 days old) of development of the gall, the antioxidant capacity was high when compared to that of the control leaf, and it indicated the presence of antioxidant groups like phenolic compounds. The accumulation of phenolic compounds and the initial ROS signaling mechanisms regulate the IAA oxidase activity. Blocking of IAA oxidase activity would help to increase local accumulation of auxin in target tissues that would enhance the dedifferentiation of tissues [20, 26]. The dedifferentiated tissues undergo hypertrophy and hyperplasia conditions. In the initial stage only, continuous proliferation of tissue occurred, but in the 2nd stage of development, high-level cascade expression of ROS signaling mechanisms was evident as clearly proved through the evaluation of the antioxidants. The methanolic extracts of the 1st and 2nd gall stages presented slight variations in their antioxidant activity. Cellular necrosis was found to start in the 2nd stage (10–13 days old galls) onwards, because the high-level activity of ROS cascade and the antioxidant capacity of 2^nd ^stage gall being an IC_50_ value of 15.21 μg/ml and that of the control leaf showed significant variation (16.69 μg/ml). The continuous cascade action of ROS could lead to hypersensitive responses (because low level of free-radical scavenging activity) and subsequently to cellular necrosis [10, 20, 26–29].

Subsequent to the 2^nd ^stage development, the antioxidant capacity of the gall appeared high, indicating the high concentration of phenolic accumulation, to prevent the ROS stress and it inhibited the cellular necrosis rates. In the 3rd stage of development, the gall showed highest antioxidant capacity by recording a value of 12.81 μg/ml and this IC_50_ rate was almost similar to that of the positive control ascorbic acid (11.05 μg/ml). In the final stage of development (4^th ^stage: 40–43 days old gall), highest ROS inhibition capacity (10.89 μg/ml) was recorded and the IC_50_ rate was higher than that of the positive control ascorbic acid. The final stage gall was clearly proved as the senescent stage with secondary metabolite accumulations, and inhibition of ROS cascade system, the latter could be in direct response to the IAA oxidation. The IAA oxidation limits the cellular proliferation rates and dedifferentiation of cells. The phenolic contents infact are secondary metabolites produced by plants as defensive compounds against herbivory. The redox properties of phenolic compounds would play vital roles in inhibition of lipoxygenase, chelating transitional metals and free radical scavenging [28, 30]. The phenolic compounds would act as effective hydrogen donors, thereby possessing good antioxidant capacity. The total phenolic concentration in the leaf gall of *P. pinnata* induced by *A. pongamiae* showed statistically significant variations between the control and middle-aged mature leaf galls [24, 30]. We have concluded that, during the feeding time (mite infestation), initially the free-radical production is high and the phenolic content is low, so there is a risk of cell damage in the tissue system, while after some time (Gall 3^rd^ stage), the host is recognized as having herbivore attacks and the plant generates secondary metabolites (phenolics compound is the main one), these metabolic compounds scaveng the free radicals and directly inhibit the ROS cascade mechanisms. Therefore, in the final stage, the phenolics accumulation being high in infested areas, it will avoid the further feeding and cellular damages caused by herbivores and also by ROS cascade mechanisms [30]. The results obtained during the present investigation on the antioxidant capacity of galls in different developmental stages showed that the phenolic and flavonoid contents were higher in the polar extracts (methanol) and subsequently, the extract possessed higher antioxidant potential also. Hence it is a clear evidence that the polar phenolics are fundamental for free radical scavenging activity as suggested earlier [14] and the present observation on the antioxidant activity could be used for promoting ethnobotanical approaches.

During the present study, 21 elements were considered for detailed analysis, and which were found distributed in the inner and outer regions of the gall tissues as well as in vascular bundle and non-vascular bundle regions of the control leaves. Of these, C, N, O, P, Hg, I, Cl, K, Ca and Mn showed positive correlation up to the 3rd stage of development of the gall. Some metals like Fe, Co, Cu, Zn, Na, Mg, Se and Al showed irregular distribution patterns. These data helped to understand the nutritional status of the inner region of the gall and their functions. In the initial stage, the concentrations of N, O, K and Mn were found higher in the outer gall region. The initial stage of development, being the time of active proliferation, necessitated the requirement for more nutrients to support normal cell cycle and hence this could be the reason for the high concentration of mobile nutrients (N, P, K, Mg, Cl, Zn and Mo) in the initial stage of development of the gall as observed during the study in the outer gall region. However, when the gall reached the second stage of development, the mobile nutrients were found moved from the outer region to the inner region, up to the 3rd stage of development of the gall. The 2^nd^ to 3^rd^ stages of gall were recognized as highly active stages, as these stages revealed the presence of more nutrients in the inner regions and their mobile nutrient concentration was found reduced when compared to those of the initial stages of the gall. Results of elemental complex analysis enabled to record clear changes in the source-sink status of the gall [19, 20, 31]. Significant variation was observed in the macro and micronutrient composition of the normal and galled leaf tissues [19]. No previous study has measured the cecidogenic nutrient profile, and determined its importance. Similar to insect galls, the mite gall often serves as a sink only in the initial stages of growth and nutrient flow has reduced reaching the senescent stage. The Hg (mercury) concentration is high at the senescent stage, typically with a high accumulation of Hg harmful to plants and herbivores. After the 3^rd^ stage, the mite feeding was reduced and the mite moved in to the new leaf field. The mercury deposition changes cell permeability, removes essential oils and also affects the light and dark reactions of photosynthesis [32]. Improper distribution of photosynthetic pigments has been reported in histochemical analysis (Fig. 6d), with Hg accumulation being the main factor behind the reduction of photosynthetic activity of gall [10, 13, 30, 32]. Not only Hg, Iodine (I) also increased in the senescent stage of gall, iodine is usually not necessary for land plants, iodine actually plays a vital role in growth, antioxidant activity and the stress tolerance response of host plants, and iodine interacts with mineral elements can be either synergistic or antagonistic [33]. Further analysis is required to answer the exact reason behind the accumulation of selected elements in the senescent stage of the gall.

Uninfested and mite infested leaf surfaces showed the presence of many organic compounds which produced complex spectra showing many absorption bands with varying intensity ranges. At the present level, understanding of all spectral peaks and their corresponding specific class of compounds was not possible. However, studies on the major spectral peak variations and corresponding functional group analysis were done and the IR spectrum analysis of control and various developmental stages of the gall showed some unique spectrum absorption peaks. Formation of low intensity absorption peaks also was observed in the uninfested samples and the galls in different developmental stages of development. Because the absorption range between 1650 and 1500 cm^− 1^ is an indicator of physiological stress [16], it will help to understand the alterations that occurred in the functional groups of the marker regions and could be used for molecular tagging. Each stage of development has specific IR spectral profiling, indicated as their cell signaling mechanisms and often correlated with the physiological responses of cells or tissue. This is the first attempt to use the ATR-FTIR technique to characterize the minute cellular impacts of biotic and abiotic stress during the development of a gall, and further studies will be needed to address the functional properties of the functional groups identified at each developmental stage. The bio- spectroscopy techniques are the important tools for non-invasive optical tissue diagnosis and it is used for detecting a wide variety of pathological states [16]. The ATR analysis of galled and non-galled leaves represents a novel tool, as far as the galls are concerned. Nowadays the ATR techniques are widely used for tracking the molecular changes, and hence in the present study it was used to help in understanding the level and variation of functional groups at different stages of gall development. These results can be used for further molecular genomic level of gall expressions.

## Conclusions

Considering the economic utility of the plant and the highly complex and intricate host-plant mite interaction between the gall mite, *A. pongamiae* and its host *P. pinnata* (L.) the present study was selected to analyze the sequences and processes involved in gall formation, through extensive studies on gall morphology, histology, histochemistry, antioxidant properties, elemental analysis and Vibrational Bio-spectrum analysis. The study was conducted during the period of March to June, 2018, considering the ease of availability of the mite in sufficient numbers from field condition, being the most favorable period for gall induction and peak population build up by the mite. During the unfavorable season, the mites hide under the bark and internodal regions, and with the onset of favorable condition they would migrate from the crevices/underneath of barks/intermodal regions of shoots to reach the young leaves for sucking up the plant sap/making the isolated niche/cecidia. Subsequently, these mites would find a suitable site on the leaf lamina for gall induction, and while sucking up the plant sap from the abaxial surface of leaf lamina, they inject saliva into the abaxial epidermis of leaves and the injected saliva would play a very vital role in triggering development of galls [5, 20, 25, 34]. Thus, the mites would trigger the cellular/molecular/genomic mechanism for gallogenesis. The gall development was found to include three major phases namely, initiation, growth and maturation [5, 20]. Each and every phase of gall development was unique in having particular characteristic structural organization.

The gall development was found completed within 40–45 days, and hence the gall should be considered as limited neoplastic growth. Data gained through ultra-structural histological studies revealed the cellular transformation in every stage of cecidogenesis, confirming the first 24–48 h of gall development as the crucial step of cecidogenesis, based on the sudden histological, vibration variation occurred in the initial stage. The significant variation showed in source-sink elemental analysis; which formed the first attempt to reveal the presence of 21 elements, following source-sink pattern of distribution in cecidia. After the 45th day, the mites completely stopped their feeding activity, and came out from the matured galls in search of new leaves of the host plant for making new gall/isolated niche on the host. Upon detection of a suitable area for development of new galls, the process of cecidogenesis would be initiated again. This study dealt with the classical aspects of gall formations, and the sequential comparative study will contribute to the development of cecidiology branch, as well as to understanding the ecology and evolution of galls.

### Limitations of the works

*P. pinnata* leaf gall is a limited neoplastic growth type, because abnormal neoplastic cells were found in the gall tissue and their growth has been completed within 40–45 days. In this study, we selected four different growth stages of leaf gall, such as the first stage (1–3 days old gall), the second stage (10–13 days old gall), the third stage (25–27 old days), and the fourth stage (40–43 old stage). If the group selection is wrong, all results would be wrong, because the histology, histochemical profile, nutrient profile, source-sink status, antioxidant activity etc. are all correlated with the age of the gall and also age of the leaf. Grouping of these galls only using the morphology method (shape and size of the gall) is very difficult, because the gall size and shape are directly correlated with the population density of the mites and their feeding activity as well as the age of the leaf (young / middle aged / matured leaf). In the study, the gall age selection is based on the shape (structural complexity), outer waxy cuticular thickening with epidermal hair structure, internal histological arrangements and the presence and amount of pigment deposition in the inner gall tissue and possession of erineal hairs. Most of the gall selection criteria are dependent on a person’s experience; it is very difficult to identify the age of the gall without proper experience in gall morphology, histology, pigment characters. To overcome these problems (selection or grouping of different gall stages), we suggested a new method i.e. ATR-FTIR analysis, IR analysis is a highly sensitive method and inexpensive, based on this method we can determine the age of the galls. The genomic expression profile and their variations can be used as prominent tool for assessing the age of the gall in future studies. Another important problem is the antioxidant activity analysis of each stages of the galls, in this study we only reported the DPPH free radical scavenging efficacy of each stage of the gall. This analysis only provides a rough overview of the antioxidative efficacy of each stages of the gall. Based on this analysis, one thing we can confirm is that each stage has a specific antioxidative profile which is directly correlated with the metabolites present at each stage of the gall. More antioxidant activity assays (such as FRAP, ABTS, etc.) needed to understand the actual antioxidant activity of each stage of the gall. Because each stage of the gall contained a wide variety of antioxidant compounds, each of the antioxidant molecule displayed a different degree of free-radical scavenging activity. Use of different antioxidant assay would help to understand the antioxidant efficacy of each stage of the gall, which will help to explain the hypersensitive and oxidative response of the gall.

## Methods

### Collection and preparation of gall samples

During the course of the present study, galled and ungalled leaves (*N* = 30) were collected randomly from different branches of *P. pinnata* L. trees growing in the Calicut University Campus, Malappuram (Dt.) of Kerala, India (identification of plant specimen by Dr. A. K. Pradeep, Asst. Professor, Department of Botany, University of Calicut). The collected samples were put in polythene bags, sealed and brought to the laboratory for subsequent studies. The galls were dissected with a sharp blade and examined under a Labovision KS z0850 Stereo-Zoom microscope, to observe mite population. Galls with parasitic animals, predators, or pathogenic infections etc. were totally discarded and not considered for subsequent analysis.

### Selection of galls in different developmental stages

Since the leaf galls induced by *A. pongamiae* attained maturity within 40–45 days (no further development), the galls selected for the present study were grouped into four major categories [5, 35]. The first stage category comprised of the galls in the initial stage of induction, representing 1–3 days old (1–2 mm length); the second stage category included 10–13 days of old galls (3–6 mm length); the third stage category was assigned to galls within 25–27 days old (0.8–1 cm length);and galls in 40–43 days old (1–1.5 cm length) were selected as the fourth stage/mature stage category. The above selection of developmental stages was done not only based on the gall size, but also on various other factors such as their shape (structural complexity), outer waxy cuticular thickening with epidermal hair structure, internal histological arrangements and the presence and amount of pigment deposition in the inner gall tissue and possession of erineal hairs.

### Histological analysis of leaf galls through field emission scanning electron microscopic (FE-SEM) studies

Histological analysis was carried out during the present study in order to understand the pattern of the cellular/tissue level organization of the leaf galls of *P. pinnata* induced by *A. pongamiae*. Freshly collected galls in different stages of development and mature middle-aged control leaves were considered for the analysis. Hand-made transverse and cross sections of the ungalled leaf as well as galls in different stages of development were made using a sharp blade (*n* = 30). The materials were fixed in 2.5% glutaraldehyde in 0.1 M phosphate buffer (pH 7.4) for 1 hour. After fixation, the plant materials were dehydrated by passing through acetone series of 70,80,90 and 100%, keeping in each for about 30 min. The dehydrated samples were dried overnight in an incubator at 25^°^C.

### Elemental analysis through energy dispersive X-ray spectroscopy (EDAX)

Considering the importance of source-sink status for determining the development of gall, the elemental complex analysis of galls in different developmental stages (*n* = 6) was carried out by using FE-SEM EDAX. Elemental analysis was performed using a ZEISS Gemini 300 FE-SEM, Germany. In total, 21 elements present in the gall samples were considered for analysis using EDAX. In the control leaves, elemental analysis was made based on vascular bundle and non-vascular tissue regions. But in the galls, owing to lack of perfect tissue organization, elemental analysis was done mainly based on the inner and outer regions of the galls.

### Histochemical characterization

Handmade thin cross sections of galls in different stages of development were prepared and subjected to histochemical analysis (*n* = 30). Detection of phenolic compounds and starch according to Johansen [8]. Reducing sugars and proteins were detected by according to Sass [36] and Backer [37] respectively.

### Determination of Antioxidative potency of the un-galled and galled leaves in different developmental stages

The leaf galls in different developmental stages as well as the ungalled leaves were collected from *P. pinnata* trees and washed thoroughly in distilled water for removing mites and debris. The cleaned samples were cut into small pieces using a sterilized sharp blade and dried at room temperature for 10–15 days in a container covered with a cotton layer. The dried samples were then powdered in a mechanical grinding machine (Mixer grinder), to facilitate effective contact between the solvent and the samples.

Using methanol, cold extraction was carried out based on the methods of Anjali Soni [38] with slight modifications (48 h at 20 °C through continuous shaking at 120 rpm in a rotary shaking incubator). The crude extracts were stored in air tight vials in a refrigerator for subsequent in-vitro antioxidant DPPH free radical scavenging assay, carried out following the methods of Blois [39]. The DPPH free radical scavenging assay was the general test used to evaluate the antioxidant capacity of plant extracts. Commonly, scavenging assay was expressed as IC_50_, (the amount of antioxidant necessary to decrease the initial concentration of DPPH by 50%). The antioxidant activity and IC_50_ values were negatively correlated, the higher IC_50_ value indicated the lower antioxidant capacity and vice versa [38]. Three independent assays were conducted and inhibition percentage (I%) of different concentrations of extracts and controls were calculated by following the equation [38] given below; IC_50_ calculated by using probit-regression analysis, this indicated how much of the extracts needed to scavenge or inhibit the 50% of free-radical.

$$ \mathrm{I}\%=\frac{\mathrm{Absorbance}\kern0.5em \mathrm{of}\kern0.5em \mathrm{control}-\mathrm{Absorbance}\kern0.5em \mathrm{of}\kern0.5em \mathrm{test}}{\mathrm{Absorbance}\kern0.5em \mathrm{of}\kern0.5em \mathrm{control}}\times \kern0.5em 100 $$

### Vibration bio-spectrum analysis – ATR-FTIR analysis

Randomly collected fresh control (ungalled) and galled leaf samples were subjected to ATR analysis following the method of Luz [15] with slight modifications. Being the preferred site for mite infestation, the abaxial/lower leaf surface was subjected to IR measurement. The leaves were placed in direct contact with ZnSe/Diamond crystal of FTIR 4600 equipped with accessory Jasco ATR Pro One device. All samples were placed in the same position to ensure optimum contact with crystal, using standard pressing mechanism of the instrument, maintaining constant pressure for all samples. Spectra reading was made thrice for each sample to avoid noise absorption peaks and average spectral details were used for later analysis. The spectra were collected over 4000 to 400 cm^− 1^ range (wave numbers), with resolution 4 cm^− 1^ and total of 25 scans per each sample with three replicas.

### Pre-processing and functional group analysis of each spectrum

The pre-processing was mainly meant for removal of noise, sloping baseline correction and normalization. Pre-processing of raw data was carried out by using BioRad laboratory Know It All (R) 2018 informatics software. Functional group analysis was made by using IrAnalyze-RAMalyze Labcognitia laboratory Ltd. bioinformatics software and also by using BioRad laboratory KnowItAll(R) 2018 informatics software. Pre-processed spectral peaks of each groups used for PCA analysis to understanding the relationship between each group.

### Statistical analyses

Statistical analyses were performed based on one-way ANOVA followed by Tukey’s Multiple range test and probit-regression analysis, using statistical package SPSS 20.0 and the data were presented as mean ± SD. Principal component analysis and correlation matrix were done by using Origin V2018b software.

## Supplementary Information

**Additional file 1 **ATR analysis data. **Spectrum 1.** Control Leaf, **Spectrum 2.** Gall 1st stage, **Spectrum 3.** Gall 2nd stage, **Spectrum 4.** Gall 3rd stage, **Spectrum 5.** Gall 3rd stage – tissue around the gall regions, **Spectrum 6.** Gall 4th stage. **Table S1**. Functional group analysis – ATR-FTIR spectrum, identified functional groups and their stability. **Table S2**. Correlation matrix of elemental analysis. **Table S3**. Source-sink pattern of gallogenesis. **Figure S1.** Complex gall structure – heavy infestation. **Table S4**. Correlation matrix of ATR-FTIR analysis.

## Data Availability

Most of the data generated or analyzed during the study are included in this article and its supplementary file.

## Abbreviations

IR: Infra-red
EDAX or EDA: Energy dispersive X-ray analyzer
PCA: Principal component analysis
PC: Principal component
ATR: Attenuated total reflectance
FTIR: Fourier-transform infrared spectroscopy
IAA: Indole-3-acetic acid
ROS: Reactive oxygen species
FE-SEM: Field emission scanning electron microscope
