# Supplementary material for: Dynamics of limited neoplastic growth on Pongamia pinnata (L.) (Fabaceae) leaf, induced by Aceria pongamiae (Acari: Eriophyidae)
Source: BMC Plant Biol. 2021 Jan 2;21:1. doi: 10.1186/s12870-020-02777-7 (PMC7777452; doi:10.1186/s12870-020-02777-7)
Supplement: Supplementary file 1 — Additional file 1 ATR analysis data. Spectrum 1. Control Leaf, Spectrum 2. Gall 1st stage, Spectrum 3. Gall 2nd stage, Spectrum 4. Gall 3rd stage, Spectrum 5. Gall 3rd stage – tissue around the gall regions, Spectrum 6. Gall 4th stage. Table S1. Functional group analysis – ATR-FTIR spectrum, identified functional groups and their stability. Table S2. Correlation matrix of elemental analysis. Table S3. Source-sink pattern of gallogenesis. Figure S1. Complex gall structure – heavy infestation. Table S4. Correlation matrix of ATR-FTIR analysis. [file 12870_2020_2777_MOESM1_ESM.docx]

**Dynamics of limited neoplastic growth on *Pongamia pinnata* (L.) (Fabaceae) leaf, induced by *Aceria pongamiae* (Acari: Eriophyidae)**

P.P, Anand^*^ and N, Ramani^*^

^*^Acarology Division, Department of Zoology, University of Calicut, Kerala, India 673 635

Spectrum 1: ATR-FTIR Spectrum of Control Leaf

**%T**

Spectrum 2: ATR-FTIR Spectrum of Gall 1^st^ stage

**%T**

Spectrum 3 : ATR-FTIR Spectrum of Gall 2^nd^ stage

**%T**

Spectrum 4 : ATR-FTIR Spectrum of Gall 3^rd^ stage

**%T**

Spectrum 5 : ATR-FTIR Spectrum of Gall 3^rd^ stage – Gall around region

**%T**

Spectrum 6 : ATR-FTIR Spectrum of Gall 4^th^ stage

**%T**

**Table 1: Functional group analysis – ATR-FTIR spectrum**

**Characterization of functional groups in Initial Stage of Gall – ATR analysis**

| **Matching Functional Groups** | | | | | |
| --- | --- | --- | --- | --- | --- |
| Sl No. | Quality | Functional group | Chemical sub class | Chemical class |  |
| 1 | 100 | Aliphatic  carbodiimide | Aliphatic carbodiimide | Aliphatic Nitrile or Multiple bonded Nitrogen |  |
|  | Corresponding chemical | | | | |
|  | **Vibration** | **Start WN** | **End WN** | **Threshold** | **Priority** |
|  | C-H Bend, CH2/CH3 | 1470 | 1405 | Variable | Very high |
|  | N=C=N Stretch, Sym | 1500 | 1400 | Variable | Mandatory |
|  | N=C=N stretch, asym | 2160 | 2130 | Very strong | Mandatory |
|  | C-H stretch, Alkyl | 3000 | 2825 | Variable | Very high |
| 2 | 100 | Aliphatic Isonitrile | Aliphatic Nitrile | Aliphatic Nitrile or Multiple Bonded Nitrogen |  |
|  | Corresponding chemical | | | | |
|  | **Vibration** | **Start WN** | **End WN** | **Threshold** | **Priority** |
|  | C-H Bend, CH2/CH3 | 1485 | 1445 | Variable | Mandatory |
|  | N≡C Stretch | 2170 | 2130 | Variable | Mandatory |
|  | C-H stretch, Alkyl | 3000 | 2855 | Variable | Medium |
| 3 | 100 | Cyclic Anhydride, Five membered |  | Aliphatic Anhydride |  |
|  | Corresponding chemical | | | | |
|  | **Vibration** | **Start WN** | **End WN** | **Threshold** | **Priority** |
|  | C-O-C stretch | 950 | 900 | Strong | Mandatory |
|  | C-OC stretch | 1300 | 1220 | Variable | Mandatory |
|  | C-H Bend, Alpha CH | 1420 | 1400 | Variable | High |
|  | C=O stretch | 1805 | 1770 | Variable | Mandatory |
|  | C=O stretch | 1875 | 1825 | Variable | Mandatory |
|  | C-H Stretch, Alkyl | 3000 | 2855 | Variable | Medium |
| 4 | 100 | Metal carbonyl, Terminal + Bridging |  | Aliphatic anhydride |  |
|  | Corresponding chemical | | | | |
|  | **Vibration** | **Start WN** | **End WN** | **Threshold** | **Priority** |
|  | >C=O Metal carbonyl | 1850 | 1750 | Strong | Mandatory |
|  | C=O Metal carbonyl | 2100 | 1900 | Very strong | Mandatory |
| 5 | 100 | Metal carbonyl, Terminal + cyclopentadienyl |  | Metal Carbonyl |  |
|  | Corresponding chemical | | | | |
|  | **Vibration** | **Start WN** | **End WN** | **Threshold** | **Priority** |
|  | Ring, C-H Bend | 845 | 810 | Variable | Mandatory |
|  | C=O Metal carbonyl | 2100 | 1930 | Very strong | Mandatory |
|  | C=O Metal carbonyl | 1985 | 1890 | Very strong | Mandatory |
|  | C-H stretch, Alkyl | 2975 | 2855 | Variable | Mandatory |
| 6 | 100 | Metal carbonyl, Terminal or Metal cyano complex |  | Metal carbonyl |  |
|  | Corresponding chemical | | | | |
|  | **Vibration** | **Start WN** | **End WN** | **Threshold** | **Priority** |
|  | C=O Metal carbonyl | 2100 | 1900 | Very strong | Mandatory |
| 7 | 100 | Strained ring or activated Carbonyl | Substituent Group | side chain or substituent |  |
|  | Corresponding chemical | | | | |
|  | **Vibration** | **Start WN** | **End WN** | **Threshold** | **Priority** |
|  | C=O stretch | 1850 | 1750 | Strong | Mandatory |
|  | C-H stretch | 3090 | 2855 | Variable | Medium |
| 8 | 95 | Tetramethyl ammonium salt |  | Aliphatic amine |  |
|  | Corresponding chemical | | | | |
|  | **Vibration** | **Start WN** | **End WN** | **Threshold** | **Priority** |
|  | C-N stretch | 955 | 940 | Variable | Mandatory |
|  | C-H bend, CH3 | 1420 | 1395 | Variable | Mandatory |
|  | C-H bend, CH3 | 1500 | 1480 | Variable | Mandatory |
|  | C-H stretch, CH3 | 3030 | 2985 | Variable | Mandatory |
| 9 | 92 | Aliphatic acid or Carbonyl compound |  | Aliphatic carboxylic acid |  |
|  | Corresponding chemical | | | | |
|  | **Vibration** | **Start WN** | **End WN** | **Threshold** | **Priority** |
|  | C-H bend, CH2/CH3 | 1450 | 1380 | Variable | Mandatory |
|  | C=O stretch | 1730 | 1680 | Strong | Mandatory |
|  | O-H Bonded, Acid | 2710 | 2580 | Variable | Mandatory |
|  | C-H stretch, Alkyl | 3120 | 2855 | Variable | Mandatory |
| 10 | 92 | Aliphatic Aldehyde |  | Aliphatic aldehyde |  |
|  | Corresponding chemical | | | | |
|  | **Vibration** | **Start WN** | **End WN** | **Threshold** | **Priority** |
|  | C-H bend, CH2/CH3 | 1480 | 1410 | Variable | Mandatory |
|  | C=O Stretch | 1740 | 1715 | Strong | Mandatory |
|  | C-H Stretch, aldehyde | 2765 | 2695 | Variable | Mandatory |
|  | C-H stretch, Alkyl | 3000 | 2855 | Variable | Mandatory |
| 11 | 92 | Carboxylic acid, General |  | Aliphatic carboxylic acid |  |
|  | Corresponding chemical | | | | |
|  | **Vibration** | **Start WN** | **End WN** | **Threshold** | **Priority** |
|  | O-H bonded, OOP | 945 | 910 | Variable | Mandatory |
|  | C-H bend, CH2/CH3 | 1450 | 1380 | Variable | Mandatory |
|  | C=O stretch | 1730 | 1680 | Strong | Mandatory |
|  | O-H bonded, Acid | 2710 | 2580 | Variable | Mandatory |
|  | C-H stretch, Alkyl | 3120 | 2855 | Variable | Mandatory |
| 12 | 90 | Silanol | Silanols | Aliphatic silicon compound |  |
|  | Corresponding chemical | | | | |
|  | **Vibration** | **Start WN** | **End WN** | **Threshold** | **Priority** |
|  | Si-O stretch | 955 | 830 | Strong | Mandatory |
|  | Si-OH Deformation | 1040 | 1020 | Medium | High |
|  | Si-CH2 Def | 1250 | 1175 | Variable | Very high |
|  | C-H stretch | 2990 | 2875 | Variable | Very high |
|  | O-H stretch | 3700 | 3200 | Variable | Mandatory |
| 13 | 83 | Isothiocyanate | Aliphatic Thicocyanate | Aliphatic Nitrile or Multiple Bonded Nitrogen |  |
|  | Corresponding chemical | | | | |
|  | **Vibration** | **Start WN** | **End WN** | **Threshold** | **Priority** |
|  | N=C=S Stretch, sym | 1240 | 1100 | Variable | Mandatory |
|  | C-H Bend, CH2/CH3 | 1470 | 1405 | Variable | Very high |
|  | N=C=S Stretch, asym | 2145 | 2080 | Very strong | Mandatory |
|  | C-H Stretch, alkyl | 3000 | 2825 | Variable | Very high |
| 14 | 76 | Aliphatic Alkoxy, Methoxy |  | Aliphatic Ether |  |
|  | Corresponding chemical | | | | |
|  | **Vibration** | **Start WN** | **End WN** | **Threshold** | **Priority** |
|  | C Skeletal, Methoxy | 980 | 905 | Medium | High |
|  | C-O Stretch | 1150 | 1070 | Variable | Mandatory |
|  | CH3 Def, Sym | 1450 | 1420 | Variable | Very high |
|  | C-H stretch, Alkyl;Methoxy | 3000 | 2820 | Variable | Mandatory |
| 15 | 71 | CF3 group | Fluorine compounds | Organic Halogen compound |  |
|  | Corresponding chemicals | | | | |
|  | **Vibration** | **Start WN** | **End WN** | **Threshold** | **Priority** |
|  | C-F stretch | 1200 | 1105 | Variable | Mandatory |
|  | C-F stretch | 1280 | 1175 | Variable | Mandatory |
|  | C-F stretch | 1375 | 1260 | Variable | Mandatory |

**Characterization of functional groups in second Stage of Gall-ATR**

| Functional Groups | | | | | |
| --- | --- | --- | --- | --- | --- |
| Sl No | Quality | Functional Group | Chemical Sub Class | Chemical Class |  |
| 1 | 100 | Aliphatic Carbodiimide | Aliphatic Carbodiimide | Aliphatic Nitrile or Multiple Bonded Nitrogen |  |
|  | Corresponding chemical | | | | |
|  | **Vibration** | **Start WN** | **End WN** | **Threshold** | **Priority** |
|  | C-H Bend, CH2/CH3 | 1470 | 1405 | Variable | Very High |
|  | N=C=N Stretch, Sym | 1500 | 1440 | Variable | Mandatory |
|  | N=C=N Stretch, asym | 2160 | 2130 | Very strong | Mandatory |
|  | C-H Stretch, Alkyl | 3000 | 2825 | Variable | Very High |
| 2 | 100 | Metal Carbonyl, Terminal + Cyclopentadienyl |  | Metal Carbonyl |  |
|  | Corresponding chemicals | | | | |
|  | **Vibration** | **Start WN** | **End WN** | **Threshold** | **Priority** |
|  | Ring, C-H Bend | 845 | 810 | Variable | Mandatory |
|  | C=O Metal Carbonyl | 1985 | 1890 | Very strong | Mandatory |
|  | C=O Metal Carbonyl | 2100 | 1930 | Very strong | Mandatory |
|  | C-H Stretch, Alkyl | 2975 | 2855 | Variable | Mandatory |
| 3 | 100 | Metal Carbonyl, Terminal or Metal Cyano complex |  | Metal Carbonyl |  |
|  | Corresponding chemicals | | | | |
|  | **Vibration** | **Start WN** | **End WN** | **Threshold** | **Priority** |
|  | C=O Metal Carbonyl | 2100 | 1900 | Very strong | Mandatory |
| 4 | 93 | Aliphatic Acid or carbonyl compound |  | Metal Carbonyl |  |
|  | Corresponding chemicals | | | | |
|  | **Vibration** | **Start WN** | **End WN** | **Threshold** | **Priority** |
|  | C-H Bend, CH2/CH3 | 1450 | 1380 | Variable | Mandatory |
|  | C=O stretch | 1730 | 1680 | Strong | Mandatory |
|  | O-H bonded, Acid | 2710 | 2580 | Variable | Mandatory |
|  | C-H stretch, Alkyl | 3120 | 2855 | Variable | Mandatory |
| 5 | 93 | Aliphatic ether or sulfone | Substituent Group | Side Chain or Substituent |  |
|  | Corresponding chemical | | | | |
|  | **Vibration** | **Start WN** | **End WN** | **Threshold** | **Priority** |
|  | SO2, Sulfoxy Stretch | 1150 | 1115 | Strong | Mandatory |
|  | SO2, Sulfoxy stretch | 1325 | 1275 | Strong | Mandatory |
|  | C-H Bend, CH2 | 1470 | 1415 | Variable | Mandatory |
|  | C-H Stretch, Alkyl | 3000 | 2855 | Variable | Mandatory |
| 6 | 93 | Carboxylic acid, General |  | Aliphatic carboxylic acid |  |
|  | Corresponding chemical | | | | |
|  | **Vibration** | **Start WN** | **End WN** | **Threshold** | **Priority** |
|  | O-H Boned, OOP | 945 | 910 | Variable | Mandatory |
|  | C-H Bend, CH2/CH3 | 1450 | 1380 | Variable | Mandatory |
|  | C=O Stretch | 1730 | 1680 | Strong | Mandatory |
|  | O-H Bonded, Acid | 2710 | 2580 | Variable | Mandatory |
|  | C-H Stretch, Alkyl | 3120 | 2855 | Variable | Mandatory |
| 7 | 92 | Aliphatic Aldehyde |  | Aliphatic Aldehyde |  |
|  | Corresponding chemical | | | | |
|  | **Vibration** | **Start WN** | **End WN** | **Threshold** | **Priority** |
|  | C-H Bend, CH2/CH3 | 1480 | 1410 | Variable | Mandatory |
|  | C=O Stretch | 1740 | 1715 | Strong | Mandatory |
|  | C-H Stretch, Aldehyde | 2765 | 2695 | Variable | Mandatory |
|  | C-H stretch, Alkyl | 3000 | 2855 | Variable | Mandatory |
| 8 | 87 | Primary Alcohol |  | Aliphatic Alcohol |  |
|  | Corresponding chemical | | | | |
|  | **Vibration** | **Start WN** | **End WN** | **Threshold** | **Priority** |
|  | C-O Stretch | 1085 | 1000 | Variable | Mandatory |
|  | O-H Def | 1400 | 1300 | Strong | Mandatory |
|  | C-H Bend, CH2/CH3 | 1475 | 1415 | Variable | High |
|  | C-H Stretch, Alkyl | 2975 | 2845 | Variable | High |
|  | O-H Stretch | 3450 | 3225 | Variable | Mandatory |
| 9 | 83 | Isothiocyanate | Aliphatic Thiocyanate | Aliphatic Nitrile or Multiple Bonded Nitrogen |  |
|  | Corresponding chemical | | | | |
|  | **Vibration** | **Start WN** | **End WN** | **Threshold** | **Priority** |
|  | N=C=S stretch, Sym | 1240 | 1100 | Variable | Mandatory |
|  | C-H Bend, CH2/CH3 | 1470 | 1405 | Variable | Very high |
|  | N=C=S Stretch, Asym | 2145 | 2080 | Very strong | Mandatory |
|  | C-H stretch, Alkyl | 3000 | 2825 | Variable | Very High |
| 10 | 80 | Hydroxy, Possibly 1,2-Diol |  | Aliphatic Alcohol |  |
|  | Corresponding chemical | | | | |
|  | **Vibration** | **Start WN** | **End WN** | **Threshold** | **Priority** |
|  | C-O stretch | 1070 | 1020 | Variable | Mandatory |
|  | O-H Def | 1400 | 1300 | Variable | Mandatory |
|  | C-H Stretch, CH2 | 2975 | 2840 | Variable | High |
|  | O-H Stretch | 3450 | 3300 | Variable | Mandatory |
| 11 | 80 | Silanol | Silanols | Aliphatic silicon compound |  |
|  | Corresponding chemical | | | | |
|  | **Vibration** | **Start WN** | **End WN** | **Threshold** | **Priority** |
|  | Si-O stretch | 955 | 830 | Strong | Mandatory |
|  | Si-OH Deformation | 1040 | 1020 | Medium | High |
|  | Si-CH2 Def | 1250 | 1175 | Variable | Very high |
|  | O-H Stretch | 3700 | 3200 | Variable | Mandatory |
| 12 | 77 | Aliphatic Alkoxy, Methoxy |  | Aliphatic Ether |  |
|  | Corresponding chemical | | | | |
|  | Vibration | Start WN | End WN | Threshold | Priority |
|  | C Skeletal, Methoxy | 980 | 905 | Medium | High |
|  | C-O Stretch | 1150 | 1070 | Variable | Mandatory |
|  | CH3 def, sym | 1450 | 1420 | Variable | Very high |
|  | C-H Stretch,Alkyl;Methoxy | 3000 | 2820 | Variable | Mandatory |
| 13 | 72 | CF3 group | Fluorine compounds | Organic Halogen compounds |  |
|  | Corresponding chemicals | | | | |
|  | **Vibration** | **Start WN** | **End WN** | **Threshold** | **Priority** |
|  | C-F stretch | 1200 | 1105 | Variable | Mandatory |
|  | C-F stretch | 1280 | 1175 | Variable | Mandatory |
|  | C-F stretch | 1375 | 1260 | Variable | Mandatory |
|  |  |  |  |  |  |

**Characterization of functional groups in third Stage of Gall - ATR**

| Functional Groups | | | | | |
| --- | --- | --- | --- | --- | --- |
| Sl No | Quality | Functional Group | Chemical sub class | Chemical class |  |
| 1 | 100 | Aliphatic Anhydride – Carbonyl compound |  | Aliphatic Anhydride |  |
|  | Corresponding chemical | | | | |
|  | **Vibration** | **Start WN** | **End WN** | **Threshold** | **Priority** |
|  | C-O Stretch | 1075 | 1005 | Variable | Mandatory |
|  | C-H Bend, CH2/CH3 | 1470 | 1425 | Variable | Very high |
|  | C=O stretch | 1785 | 1740 | Variable | Mandatory |
|  | C=O stretch | 1850 | 1805 | Variable | Mandatory |
|  | C-H stretch, Alkyl | 3000 | 2855 | Variable | High |
| 2 | 100 | Aliphatic Isocyanate | Aliphatic Cyanate | Aliphatic Nitrile or Multiple Bonded Nitrogen |  |
|  | Corresponding chemical | | | | |
|  | **Vibration** | **Start WN** | **End WN** | **Threshold** | **Priority** |
|  | NCO Def | 645 | 590 | Variable | Mandatory |
|  | C-N Bend | 890 | 830 | Variable | Mandatory |
|  | NCO Stretch, sym | 1440 | 1360 | Variable | Mandatory |
|  | C-H Bend, CH2/CH3 | 1470 | 1445 | Variable | Mandatory |
|  | NCO Stretch, Asym | 2295 | 2250 | Very strong | Mandatory |
|  | C-H stretch, Alkyl | 3000 | 2825 | Variable | Medium |
| 3 | 100 | Cyclic Anhydride, Five membered |  | Aliphatic Anhydride |  |
|  | Corresponding chemical | | | | |
|  | **Vibration** | **Start WN** | **End WN** | **Threshold** | **Priority** |
|  | C-O-C stretch | 950 | 900 | Strong | Mandatory |
|  | C-O-C stretch | 1300 | 1220 | Variable | Mandatory |
|  | C-H bend, Alpha CH | 1420 | 1400 | Variable | High |
|  | C=O stretch | 1805 | 1770 | Variable | Mandatory |
|  | C=O stretch | 1875 | 1825 | Variable | Mandatory |
|  | C-H stretch, Alkyl | 3000 | 2855 | Variable | Medium |
| 4 | 100 | Hydro Siloxane | Sily Ethers | Aliphatic Silicon compound |  |
|  | Corresponding chemical | | | | |
|  | Vibration | Start WN | End WN | Threshold | Priority |
|  | Si-O | 895 | 845 | Strong | Mandatory |
|  | Alkoxy C-O | 1100 | 1050 | Strong | Mandatory |
|  | Si-H stretch | 2200 | 2100 | Variable | Mandatory |
|  | C-H stretch | 3000 | 2940 | Variable | Medium |
| 5 | 100 | Metal Carbonyl, Terminal + Bridging |  | Metal Carbonyl |  |
|  | Corresponding chemical | | | | |
|  | **Vibration** | **Start WN** | **End WN** | **Threshold** | **Priority** |
|  | >C=O Metal carbonyl | 1850 | 1750 | Strong | Mandatory |
|  | C=O Metal Carbonyl | 2100 | 1900 | Very strong | Mandatory |
| 6 | 100 | Metal carbonyl, Terminal + Cyclopentadienyl |  | Metal carbonyl |  |
|  | Corresponding chemical | | | | |
|  | **Vibration** | **Start WN** | **End WN** | **Threshold** | **Priority** |
|  | Ring, C-H Bend | 845 | 810 | Variable | Mandatory |
|  | C=O Metal Carbonyl | 1985 | 1890 | Very strong | Mandatory |
|  | C=O Metal Carbonyl | 2100 | 1930 | Very strong | Mandatory |
|  | C-H stretch, Alkyl | 2975 | 2855 | Variable | Mandatory |
| 7 | 100 | Metal carbonyl, Terminal + Metal Cyano complex |  | Metal Carbonyl |  |
|  | Corresponding chemical | | | | |
|  | **Vibration** | **Start WN** | **End WN** | **Threshold** | **Priority** |
|  | C=O Metal Carbonyl | 2100 | 1900 | Very strong | Mandatory |
| 8 | 100 | Strained Ring or Activated Carbonyl | Substituent Group | Side Chain or Substituent |  |
|  | Corresponding chemical | | | | |
|  | **Vibration** | **Start WN** | **End WN** | **Threshold** | **Priority** |
|  | C=O stretch | 1850 | 1750 | Strong | Mandatory |
|  | C-H stretch | 3090 | 2855 | Variable | Medium |
| 9 | 99 | Aliphatic Carbodiimide | Aliphatic Carbodiimide | Aliphatic Nitrile or Multiple Bonded Nitrogen |  |
|  | Corresponding Chemical | | | | |
|  | **Vibration** | **Start WN** | **End WN** | **Threshold** | **Priority** |
|  | C-H Bend, CH2/CH3 | 1470 | 1405 | Variable | Very High |
|  | N=C=N stretch, sym | 1500 | 1440 | Variable | Mandatory |
|  | N=C=N stretch, asym | 2160 | 2130 | Very strong | Mandatory |
|  | C-H stretch, Alkyl | 3000 | 2825 | Variable | Very high |
| 10 | 99 | Aliphatic cyanate | Aliphatic cyanate | Aliphatic Nitrile or Multiple Bonded Nitrogen |  |
|  |  | | | | |
|  | **Vibration** | **Start WN** | **End WN** | **Threshold** | **Priority** |
|  | COCN stretch | 1140 | 1080 | Variable | Mandatory |
|  | C-H Bend, CH2/CH3 | 1470 | 1435 | Variable | Mandatory |
|  | OCN stretch | 2265 | 2240 | Strong | Mandatory |
|  | C-H stretch, alkyl | 3000 | 2855 | Variable | High |
| 11 | 99 | Aliphatic Isonitrile | Aliphatic Nitrile | Aliphatic Nitrile or multiple bonded Nitrogen |  |
|  | Corresponding chemical | | | | |
|  | **Variable** | **Start WN** | **End WN** | **Threshold** | **Priority** |
|  | C-H Bend, CH2/CH3 | 1485 | 1445 | Variable | Mandatory |
|  | N≡C Stretch | 2170 | 2130 | Variable | Mandatory |
|  | C-H stretch, Alkyl | 3000 | 2855 | Variable | Medium |
| 12 | 94 | Tetramethyl ammonium salt |  | Aliphatic Amine |  |
|  | Corresponding chemical | | | | |
|  | **Vibration** | **Start WN** | **End WN** | **Threshold** | **Priority** |
|  | C-N stretch | 955 | 940 | Variable | Mandatory |
|  | C-H Bend, CH3 | 1420 | 1395 | Variable | Mandatory |
|  | C-H Bend, CH3 | 1500 | 1480 | Variable | Mandatory |
|  | C-H stretch, CH3 | 3030 | 2985 | Variable | Mandatory |
| 13 | 93 | Aliphatic tert-Amino acid |  | Aliphatic Amino Acid |  |
|  | Corresponding chemical | | | | |
|  | **Vibration** | **Start WN** | **End WN** | **Threshold** | **Priority** |
|  | C-H bend | 1425 | 1390 | Variable | Mandatory |
|  | C-H bend, CH2/CH3 | 1470 | 1430 | Variable | Mandatory |
|  | N-H Bend | 1530 | 1500 | Variable | Mandatory |
|  | C=O stretch | 1740 | 1640 | Strong | Mandatory |
|  | C-H stretch, Alkyl | 3000 | 2855 | Variable | Mandatory |
|  | N-H stretch, Bonded | 3300 | 3100 | Variable | Mandatory |
| 14 | 91 | Aliphatic acid or Carbonyl compound |  | Aliphatic Carboxylic acid |  |
|  | Corresponding chemical | | | | |
|  | **Vibration** | **Start WN** | **End WN** | **Threshold** | **Priority** |
|  | C-H Bend, CH2/CH3 | 1450 | 1380 | Variable | Mandatory |
|  | C=O stretch | 1730 | 1680 | Strong | Mandatory |
|  | O-H Bonded, Acid | 2710 | 2580 | Variable | Mandatory |
|  | C-H stretch, Alkyl | 3120 | 2855 | Variable | Mandatory |
| 15 | 91 | Aliphatic Sulfonate or sulfate | Aliphatic Sulfoxy-compound | Aliphatic Sulfur compound |  |
|  | Corresponding chemical | | | | |
|  | **Vibration** | **Start WN** | **End WN** | **Threshold** | **Priority** |
|  | S-O stretch,Sym | 830 | 700 | Variable | Very high |
|  | S-O stretch | 1010 | 900 | Strong | Mandatory |
|  | S=O stretch, Sym | 1210 | 1150 | Strong | Mandatory |
|  | S=O stretch, asym | 1420 | 1340 | Strong | Mandatory |
|  | C-H Bend, CH2/CH3 | 1480 | 1410 | Variable | Mandatory |
|  | C-H stretch, Alkyl | 3000 | 2855 | Variable | High |
| 16 | 91 | Carboxylic acid, General |  | Aliphatic Carboxylic acid |  |
|  | Corresponding chemical | | | | |
|  | **Vibration** | **Start WN** | **End WN** | **Threshold** | **Priority** |
|  | O-H Bonded, OOP | 945 | 910 | Variable | Mandatory |
|  | C-H Bend, CH2/CH3 | 1450 | 1380 | Variable | Mandatory |
|  | C=O Stretch | 1730 | 1680 | Strong | Mandatory |
|  | O-H Bonded,Acid | 2710 | 2580 | Variable | Mandatory |
|  | C-H Stretch,Alkyl | 3120 | 2855 | Variable | Mandatory |
| 17 | 91 | Linear Bromo compound | Aliphatic Brominated compound | Aliphatic Halogen compound |  |
|  | Corresponding chemical | | | | |
|  | **Vibration** | **Start WN** | **End WN** | **Threshold** | **Priority** |
|  | C-H wag, Halogen Substituted | 1245 | 1225 | Variable | Mandatory |
|  | C-H Bend, CH3 | 1380 | 1370 | Variable | Medium |
|  | C-H def, Alpha CH | 1440 | 1385 | Variable | Mandatory |
|  | C-H bend, CH2/CH3 | 1465 | 1425 | Medium | Medium |
|  | C-H stretch, Alkyl | 3030 | 2900 | Variable | High |
| 18 | 90 | Aliphatic Sulfite | Aliphatic Sulfoxy-compound | Aliphatic sulfur compound |  |
|  | Corresponding chemical | | | | |
|  | **Vibration** | **Start WN** | **End WN** | **Threshold** | **Priority** |
|  | S-O, stretch | 740 | 700 | Variable | Mandatory |
|  | S=O/S-O, Sulfoxy stretch | 1040 | 980 | Strong | Mandatory |
|  | S=O, Sulfoxy stretch | 1215 | 1180 | Strong | Mandatory |
|  | C-H Wag | 1375 | 1315 | Variable | Medium |
|  | C-H def | 1480 | 1440 | Variable | Medium |
|  | C-H Stretch, Alkyl | 3000 | 2855 | Variable | Medium |
| 19 | 90 | Carbonyl, Possibly 2-Hydroxy Acid |  | Aliphatic Carboxylic acid |  |
|  | Corresponding chemical | | | | |
|  | **Vibration** | **Start WN** | **End WN** | **Threshold** | **Priority** |
|  | O-H bonded | 950 | 880 | Variable | Mandatory |
|  | C-H Bend, CH2/CH3 | 1475 | 1400 | Variable | Mandatory |
|  | C=O stretch, Conjugated | 1720 | 1680 | Strong | Mandatory |
|  | O-H bonded, Acid | 2710 | 2580 | Variable | Mandatory |
|  | C-H stretch, Alkyl | 2990 | 2900 | Variable | Mandatory |
|  | O-H stretch, 2-Hydroxy | 3500 | 3380 | Variable | Mandatory |
| 20 | 90 | Hydroxy, Possibly 1,2-Diol |  | Aliphatic Alcohol |  |
|  | Corresponding Chemical | | | | |
|  | **Vibration** | **Start WN** | **End WN** | **Threshold** | **Priority** |
|  | C-O Stretch | 1070 | 1020 | Variable | Mandatory |
|  | O-H Def | 1400 | 1300 | Variable | Mandatory |
|  | C-H Bend, CH2/CH3 | 1475 | 1445 | Variable | Very High |
|  | C-H stretch, CH2 | 2975 | 2840 | Variable | High |
|  | O-H Stretch | 3450 | 3300 | Variable | Mandatory |
| 21 | 90 | Methyl Mercapto | Aliphatic Thioether | Aliphatic Thiocompound |  |
|  | Corresponding Chemical | | | | |
|  | **Vibration** | **Start WN** | **End WN** | **Threshold** | **Priority** |
|  | C-S Stretch | 750 | 685 | Variable | Mandatory |
|  | Methyl CH3-S- | 1000 | 960 | Variable | Mandatory |
|  | C-H Bend, CH3 | 1340 | 1300 | Variable | Very high |
|  | C-H Bend, CH3 | 1455 | 1405 | Variable | Very High |
|  | C-H Stretch, CH3-S- | 3030 | 2855 | Variable | Very high |
| 22 | 90 | Tertiary Alcohol |  | Aliphatic Alcohol |  |
|  | Corresponding chemical | | | | |
|  | **Vibration** | **Start WN** | **End WN** | **Threshold** | **Priority** |
|  | C-O Def | 800 | 750 | Variable | Mandatory |
|  | C-O Stretch | 1210 | 1100 | Variable | Mandatory |
|  | O-H Def | 1400 | 1320 | Variable | Mandatory |
|  | C-H Bend, CH2/CH3 | 1480 | 1410 | Variable | Mandatory |
|  | C-H stretch, Alkyl | 3000 | 2855 | Variable | Mandatory |
|  | O-H stretch | 3450 | 3255 | Variable | Mandatory |
| 23 | 89 | Aliphatic Aldehyde |  | Aliphatic Aldehyde |  |
|  | Corresponding Chemical | | | | |
|  | **Vibration** | **Start WN** | **End WN** | **Threshold** | **Priority** |
|  | C-H Bend, CH2/CH3 | 1480 | 1410 | Variable | Mandatory |
|  | C=O Stretch | 1740 | 1715 | Strong | Mandatory |
|  | C-H Stretch, Aldehyde | 2765 | 2695 | Variable | Mandatory |
|  | C-H stretch, Alkyl | 3000 | 2855 | Variable | Mandatory |
| 24 | 89 | Aliphatic Carboxylate, Possibly Trifluroacetate |  | Aliphatic Carboxylase |  |
|  | Corresponding Chemical | | | | |
|  | **Vibration** | **Start WN** | **End WN** | **Threshold** | **Priority** |
|  | C-O Bend, Carboxylate | 735 | 715 | Variable | Mandatory |
|  | C-O Bend, Carboxylate | 805 | 775 | Variable | Mandatory |
|  | Skeletal | 860 | 835 | Variable | Mandatory |
|  | C-F Stretch | 1215 | 1170 | Strong | Mandatory |
|  | C-O Stretch, Carboxylate | 1465 | 1440 | Medium | Mandatory |
|  | C-O Stretch, Carboxylate | 1695 | 1650 | Strong | Mandatory |
| 25 | 89 | Aliphatic Ether or Sulfonate salt | Substituent Group | Side chain or Substituent |  |
|  | Corresponding chemical | | | | |
|  | **Vibration** | **Start WN** | **End WN** | **Threshold** | **Priority** |
|  | SO2, Sulfoxy stretch | 1065 | 1030 | Variable | Mandatory |
|  | SO2,Sulfoxy stretch | 1205 | 1160 | Strong | Mandatory |
|  | C-H Bend, CH2 | 1470 | 1415 | Variable | Mandatory |
|  | C-H Stretch, Alkyl | 3000 | 2855 | Variable | Mandatory |
| 26 | 89 | Aliphatic Ether or Sulfone | Substituent Group | Side Chain or Substituent |  |
|  | Corresponding Chemical | | | | |
|  | **Vibration** | **Start WN** | **End WN** | **Threshold** | **Priority** |
|  | SO2 , Sulfoxy Stretch | 1150 | 1115 | Strong | Mandatory |
|  | SO2, Sulfoxy stretch | 1325 | 1275 | Strong | Mandatory |
|  | C-H Bend, CH2 | 1470 | 1415 | Variable | Mandatory |
|  | C-H Stretch, Alkyl | 3000 | 2855 | Variable | Mandatory |
| 27 | 89 | Aliphatic Ether or Sulfoxide | Substituent Group | Side chain or Substituent |  |
|  | Corresponding Chemical | | | | |
|  | **Vibration** | **Start WN** | **End WN** | **Threshold** | **Priority** |
|  | C-S=O, Sulfoxy stretch | 1060 | 1020 | Strong | Mandatory |
|  | C-H Bend, CH2 | 1470 | 1415 | Variable | Mandatory |
|  | C-H stretch, Alkyl | 3000 | 2855 | Variable | Mandatory |
| 28 | 89 | Aliphatic Mercapto Group | Substituent Group | Side Chain or Substituent |  |
|  | Corresponding chemical | | | | |
|  | **Vibration** | **Start WN** | **End WN** | **Threshold** | **Priority** |
|  | C-S stretch | 680 | 645 | Variable | Mandatory |
|  | C-S, CH2 bend | 1290 | 1220 | Variable | Mandatory |
|  | C-H Bend, CH2/CH3 | 1480 | 1410 | Variable | Mandatory |
|  | S-H stretch | 2565 | 2525 | Variable | Mandatory |
|  | C-H Stretch, Alkyl | 3000 | 2855 | Variable | Mandatory |
| 29 | 89 | Hydroxy compound | Substituent Group | Side chain or substituent |  |
|  | Corresponding chemical | | | | |
|  | **Vibration** | **Start WN** | **End WN** | **Threshold** | **Priority** |
|  | C-O Stretch | 1125 | 1000 | Strong | Mandatory |
|  | C-H Bend, CH2/CH3 | 1475 | 1415 | Variable | Mandatory |
|  | C-H Stretch, Alkyl | 2975 | 2845 | Variable | Medium |
|  | O-H Stretch | 3450 | 3225 | Variable | Mandatory |
| 30 | 89 | Silanol | Silanols | Aliphatic Silicon Compound |  |
|  | Corresponding Chemical | | | | |
|  | **Vibration** | **Start WN** | **End WN** | **Threshold** | **Priority** |
|  | Si-O Stretch | 955 | 830 | Strong | Mandatory |
|  | Si-OH Deformation | 1040 | 1020 | Medium | High |
|  | Si-CH2 Def | 1250 | 1175 | Variable | Very High |
|  | C-H Stretch | 2990 | 2875 | Variable | Very High |
|  | O-H Stretch | 3700 | 3200 | Variable | Mandatory |
| 31 | 80 | Aliphatic Alkoxy, Methoxy |  | Aliphatic Ether |  |
|  | Corresponding Chemical | | | | |
|  | **Vibration** | **Start WN** | **End WN** | **Threshold** | **Priority** |
|  | C Skeletal, Methoxy | 980 | 905 | Medium | High |
|  | C-O Stretch | 1150 | 1070 | Variable | Mandatory |
|  | CH3 def,sym | 1450 | 1420 | Variable | Very High |
|  | CH3 def, asym | 1480 | 1435 | Variable | Very High |
|  | C-H Stretch, Alkyl;Methoxy | 3000 | 2820 | Variable | Mandatory |
| 32 | 80 | Aliphatic Nitro Group |  | Aliphatic Nitro-oxy Compound |  |
|  | Corresponding Chemical | | | | |
|  | **Vibration** | **Start WN** | **End WN** | **Threshold** | **Priority** |
|  | NO2 Def | 650 | 600 | Variable | Mandatory |
|  | C-N Stretch | 990 | 920 | Variable | Very High |
|  | NO2 Stretch, Sym | 1385 | 1355 | Variable | Mandatory |
|  | C-H Bend, CH2/CH3 | 1460 | 1420 | Variable | Mandatory |
|  | NO2 Stretch,asym | 1560 | 1540 | Very strong | Mandatory |
|  | C-H Stretch, Alkyl | 3000 | 2855 | Variable | Medium |
| 33 | 80 | Metal Carbonyl, Terminal+ Aryl |  | Metal Carbonyl |  |
|  | Corresponding chemical | | | | |
|  | **Vibration** | **Start WN** | **End WN** | **Threshold** | **Priority** |
|  | Ring, C-H Bend | 685 | 655 | Variable | Mandatory |
|  | C=O Metal Carbonyl | 1890 | 1840 | Very Strong | Mandatory |
|  | C=O Metal Carbonyl | 1990 | 1920 | Very Strong | Mandatory |
| 34 | 80 | Primary Alcohol |  | Aliphatic Alcohol |  |
|  | Corresponding chemical | | | | |
|  | **Vibration** | **Start WN** | **End WN** | **Threshold** | **Priority** |
|  | C-O Stretch | 1080 | 1000 | Variable | Mandatory |
|  | O-H Def | 1400 | 1300 | Strong | Mandatory |
|  | C-H Bend, CH2/CH3 | 1475 | 1415 | Variable | High |
|  | C-H Stretch,Alkyl | 2975 | 2845 | Variable | High |
|  | O-H stretch | 3450 | 3225 | Variable | Mandatory |
| 35 | 79 | Epoxy Substituent | Substituent Group | Side Chain or Substituent |  |
|  | Corresponding Chemical | | | | |
|  | **Vibration** | **Start WN** | **End WN** | **Threshold** | **Priority** |
|  | Epoxy | 850 | 830 | Strong | Mandatory |
|  | Epoxy | 955 | 900 | Variable | Mandatory |
|  | Epoxy | 1270 | 1250 | Variable | Mandatory |
|  | C-H Stretch, Epoxy | 1415 | 1400 | Variable | Mandatory |
| 36 | 78 | Aliphatic Sulphoxide | Aliphatic Sulfoxy-Compound | Aliphatic sulfur Compound |  |
|  |  | | | | |
|  | **Vibration** | **Start WN** | **End WN** | **Threshold** | **Priority** |
|  | C-S stretch | 730 | 665 | Variable | Mandatory |
|  | S=O Stretch | 1050 | 1030 | Variable | Mandatory |
|  | C-H Def | 1480 | 1420 | Variable | Mandatory |
|  | C-H Stretch | 2990 | 2875 | Variable | Mandatory |
| 37 | 78 | CHF2 | Fluorine compounds | Organic Halogen Compounds |  |
|  | Corresponding chemical | | | | |
|  | **Vibration** | **Strat WN** | **End WN** | **Threshold** | **Priority** |
|  | CF2 wagging | 700 | 600 | Variable | High |
|  | C-F Stretch, Sym | 1125 | 1055 | Strong | Mandatory |
|  | C-F Stretch,asym | 1205 | 1105 | Strong | Mandatory |
|  | C-H Def | 1345 | 1210 | Variable | High |
|  | C-H Def | 1440 | 1350 | Variable | High |
|  | C-H Stretch | 3005 | 2975 | Variable | Mandatory |
| 38 | 77 | Linear Chloro Compound | Aliphatic chlorinated compound | Aliphatic Halogen compound |  |
|  | Corresponding chemical | | | | |
|  | **Vibration** | **Start WN** | **End WN** | **Threshold** | **Priority** |
|  | C-Cl Stretch | 740 | 650 | Variable | Mandatory |
|  | C-H wag, Chloro substituted | 1315 | 1240 | Variable | Mandatory |
|  | C-H Bend, CH3 | 1400 | 1355 | Variable | Mandatory |
|  | C-H Bend, CH2/CH3 | 1480 | 1415 | Variable | Mandatory |
|  | C-H Stretch, Alkyl | 3020 | 2920 | Strong | High |
| 39 | 74 | CF3 Group | Fluorine Compounds | Orgainc Halogen Compounds |  |
|  | Corresponding chemical | | | | |
|  | **Vibration** | **Start WN** | **End WN** | **Threshold** | **Priority** |
|  | C-F def | 770 | 650 | Weak | Very high |
|  | C-F Stretch | 1200 | 1105 | Variable | Mandatory |
|  | C-F Stretch | 1280 | 1175 | Variable | Mandatory |
|  | C-F Stretch | 1375 | 1260 | Variable | Mandatory |
| 40 | 71 | Aliphatic acid Halide, Long chain linear |  | Aliphatic acid Halide |  |
|  | Corresponding chemical | | | | |
|  | **Vibration** | **Start WN** | **End WN** | **Threshold** | **Priority** |
|  | X-C=0,C-X Stretch | 745 | 600 | Variable | High |
|  | C-C=O, C-C Stretch | 960 | 935 | Variable | Mandatory |
|  | C-C Skeletal | 1155 | 1120 | Variable | High |
|  | C-H bend, Alpha CH2 | 1210 | 1190 | Variable | Mandatory |
|  | C-H Bend, CH2/CH3 | 1470 | 1445 | Variable | Mandatory |
|  | C=O Stretch | 1825 | 1780 | Strong | Mandatory |
| 41 | 71 | Aliphatic Thiocyanate | Aliphatic Thiocyanate | Aliphatic Nitrile or Multiple Bonded Nitrogen |  |
|  | Corresponding chemical | | | | |
|  | **Vibration** | **Start WN** | **End WN** | **Threshold** | **Priority** |
|  | C-S-C | 660 | 620 | Variable | Mandatory |
|  | C-S-C | 700 | 680 | Weak | Very high |
|  | C-H bend, CH2/CH3 | 1470 | 1425 | Variable | Mandatory |
|  | S-C≡N asym Stretch | 2165 | 2140 | Variable | Mandatory |
|  | C-H stretch, Alkyl | 3000 | 2855 | Variable | Medium |
| 42 | 70 | Aliphatic Acid Halide,Linear |  | Aliphatic acid Halide |  |
|  | Corresponding Chemical | | | | |
|  | **Vibration** | **Start WN** | **End WN** | **Threshold** | **Priority** |
|  | X-C=O,C-X Stretch | 780 | 600 | Variable | Mandatory |
|  | C-C=O,C-C Stretch | 960 | 910 | Variable | Very High |
|  | C-H bend, Alpha CH2 | 1210 | 1190 | Variable | Very high |
|  | C-H bend, CH2/CH3 | 1470 | 1445 | Variable | High |
|  | C=O stretch | 1825 | 1780 | Variable | Mandatory |
|  | C-H stretch,Alkyl | 3000 | 2855 | Variable | Medium |
| 43 | 69 | Isothiocyanate | Aliphatic Thiocyanate | Aliphatic Nitrile or Multiple Bonded Nitrogen |  |
|  | Corresponding chemical | | | | |
|  | **Vibration** | **Start WN** | **End WN** | **Threshold** | **Priority** |
|  | N=C=S stretch,sym | 1240 | 1100 | Variable | Mandatory |
|  | C-H Bend, CH2/CH3 | 1470 | 1405 | Variable | Very high |
|  | N=C=S stretch, asym | 2145 | 2080 | Very strong | Mandatory |
|  | C-H Stretch, Alkyl | 3000 | 2825 | Variable | Very high |
| 44 | 67 | Phenyl substituent | Aryl Group | Aromatic compound |  |
|  | Corresponding chemical | | | | |
|  | **Vibration** | **Start WN** | **End WN** | **Threshold** | **Priority** |
|  | C-H Bend, OOP | 710 | 680 | Variable | Mandatory |
|  | C-H Bend, OOP | 745 | 725 | Variable | Mandatory |
|  | C-C Stretch, Ring | 1515 | 1485 | Variable | Mandatory |
| 45 | 64 | Possibly Methyl Siloxane or Silane | Substituent group | Side chain or Substituent |  |
|  | Corresponding chemical | | | | |
|  | **Vibration** | **Start WN** | **End WN** | **Threshold** | **Priority** |
|  | Si-C Stretch | 860 | 820 | Strong | Mandatory |
|  | Si-CH3 (C-H Bend) | 1260 | 1235 | Strong | Mandatory |
| 46 | 59 | Aliphatic tertiary |  | Aliphatic Amide |  |
|  | Corresponding chemical | | | | |
|  | **Vibration** | **Start WN** | **End WN** | **Threshold** | **Priority** |
|  | C-N stretch/C-C stretch | 1150 | 1050 | Variable | Mandatory |
|  | C-H bend | 1460 | 1410 | Variable | Mandatory |
|  | C=O stretch, Amido | 1690 | 1645 | Strong | Mandatory |
|  | C-H Stretch, Alkyl | 3000 | 2855 | Variable | Medium |

**Characterization of functional groups in fourth Stage of Gall ATR analysis**

| Functional Groups | | | | | |
| --- | --- | --- | --- | --- | --- |
| Sl NO | Quality | Functional Groups | Chemical sub class | Chemical class |  |
| 1 | 100 | Aliphatic Amine salt,Tertiary,Hydrochloride |  | Aliphatic amine |  |
|  | Corresponding chemical | | | | |
|  | **Vibration** | **Start WN** | **End WN** | **Threshold** | **Priority** |
|  | C-N stretch | 1035 | 980 | Variable | Mandatory |
|  | C-H Skeletal | 1325 | 1275 | Variable | Mandatory |
|  | C-H Bend, CH2/CH3 | 1420 | 1375 | Variable | Mandatory |
|  | C-H Bend, CH2/CH3 | 1485 | 1455 | Variable | Mandatory |
|  | N-H stretch, Bonded | 2150 | 2450 | Strong | Mandatory |
|  | N-H stretch, Bonded | 2680 | 2580 | Strong | Mandatory |
| 2 | 99 | Aliphatic anhydride- Carbonyl compound |  | Aliphatic Anhydride |  |
|  | Corresponding chemical | | | | |
|  | **Vibration** | **Start WN** | **End WN** | **Threshold** | **Priority** |
|  | C-O stretch | 1075 | 1005 | Variable | Mandatory |
|  | C-H Bend, CH2/CH3 | 1470 | 1425 | Variable | Very high |
|  | C=O Stretch | 1785 | 1740 | Variable | Mandatory |
|  | C=O Stretch | 1850 | 1805 | Variable | Mandatory |
|  | C-H Stretch, Alkyl | 3000 | 2855 | Variable | High |
| 3 | 99 | Aliphatic Carbodiimide | Aliphatic Carbodiimide | Aliphatic Nitrile or Multiple bonded Nitrogen |  |
|  | Corresponding chemical | | | | |
|  | **Vibration** | **Start WN** | **End WN** | **Threshold** | **Priority** |
|  | C-H Bend,CH2/CH3 | 1470 | 1405 | Variable | Very high |
|  | N=C=N Stretch, sym | 1500 | 1440 | Variable | Mandatory |
|  | N=C=N Stretch, Asym | 2160 | 2130 | Very strong | Mandatory |
|  | C-H Stretch,Alkyl | 3000 | 2825 | Variable | Very high |
| 4 | 99 | Aliphatic Cyanate | Aliphatic Cyanate | Aliphatic Nitrile or Multiple bonded Nitrogen |  |
|  | Corresponding chemical | | | | |
|  | **Vibration** | **Start WN** | **End WN** | **Threshold** | **Priority** |
|  | COCN stretch | 1140 | 1080 | Variable | Mandatory |
|  | C-H Bend, CH2/CH3 | 1470 | 1435 | Variable | Mandatory |
|  | OCN Stretch | 2265 | 2240 | Strong | Mandatory |
|  | C-H Stretch,Alkyl | 3000 | 2855 | Variable | High |
| 5 | Aliphatic Isonitrile | Aliphatic Nitrile |  | Aliphatic Nitrile or Multiple Bonded Nitrogen |  |
|  | Corresponding chemical | | | | |
|  | **Vibration** | **Start WN** | **End WN** | **Threshold** | **Priority** |
|  | C-H bend, CH2/CH3 | 1485 | 1445 | Variable | Mandatory |
|  | N≡C Stretch | 2170 | 2130 | Variable | Mandatory |
|  | C-H Stretch, Alkyl | 3000 | 2855 | Variable | Medium |
| 6 | 99 | Metal Carbonyl, Terminal + Bridging |  | Metal Carbonyl |  |
|  | Corresponding chemical | | | | |
|  | **Vibration** | **Start WN** | **End WN** | **Threshold** | **Priority** |
|  | >C=O Metal Carbonyl | 1850 | 1750 | Strong | Mandatory |
|  | C=O Metal carbonyl | 2100 | 1900 | Very strong | Mandatory |
| 7 | 99 | Metal Carbonyl, Terminal or Metal cyano complex |  | Metal Carbonyl |  |
|  |  | | | | |
|  | **Vibration** | **Start WN** | **End WN** | **Threshold** | **Priority** |
|  | C=O Metal carbonyl | 2100 | 1900 | Very strong | Mandatory |
| 8 | 99 | Strained Ring or Activated Carbonyl | Substituent Group | Side Chain or Substituent |  |
|  | Corresponding chemical | | | | |
|  | **Vibration** | **Start WN** | **End WN** | **Threshold** | **Priority** |
|  | C=O stretch | 1850 | 1750 | Strong | Mandatory |
|  | C-H stretch | 3090 | 2855 | Variable | Medium |
| 9 | 98 | Unsaturated Hydrocarbon, Simple Alkyne |  | Alkyne |  |
|  | Corresponding chemical | | | | |
|  | **Vibration** | **Start WN** | **End WN** | **Threshold** | **Priority** |
|  | C-H Bend, Alkyl | 1470 | 1430 | Variable | Mandatory |
|  | C≡C Stretch, Alkyne | 2140 | 2085 | Weak | Mandatory |
|  | C-H Stretch, Alkyl | 3000 | 2900 | Strong | Mandatory |
| 10 | 95 | N-Methyl Amino, Tertiary,Aliphatic |  | Aliphatic Amine |  |
|  | Corresponding Chemical | | | | |
|  | **Vibration** | **Start WN** | **End WN** | **Threshold** | **Priority** |
|  | C-N Stretch | 1200 | 1180 | Variable | Mandatory |
|  | C-N stretch | 1280 | 1260 | Variable | Mandatory |
|  | C-H Bend, Sym, CH3 | 1435 | 1365 | Variable | Mandatory |
|  | C-H Bend, asym,CH2/CH3 | 1490 | 1440 | Variable | Medium |
|  | C-H Stretch, CH3-N- | 2820 | 2765 | Variable | Medium |
|  | C-H stretch, CH2/CH3 | 3020 | 2850 | Variable | Mandatory |
|  | No Band Region | 1650 | 1570 | Very strong | Excluded |
| 11 | 94 | Aliphatic Thioether | Aliphatic Thioether | Aliphatic Thiocompound |  |
|  | Corresponding Chemical | | | | |
|  | **Vibration** | **Start WN** | **End WN** | **Threshold** | **Priority** |
|  | C-H stretch | 1000 | 950 | Variable | Mandatory |
|  | C-H/C-S, CH2 Wagging | 1285 | 1260 | Variable | Mandatory |
|  | C-H Bend, CH2 | 1380 | 1360 | Variable | Mandatory |
|  | C-H Bend, CH2/C-S | 1435 | 1410 | Variable | Mandatory |
|  | C-H Bend, CH2/CH3 | 1480 | 1440 | Variable | Mandatory |
|  | C-H stretch, Alkyl | 2870 | 2845 | Variable | Mandatory |
|  | C-H Stretch, CH2 | 2965 | 2915 | Variable | mandatory |
| 12 | 88 | Aliphatic Alkoxy, Methoxy |  | Aliphatic Ether |  |
|  | Corresponding Chemical | | | | |
|  | **Vibration** | **Start WN** | **End WN** | **Threshold** | **Priority** |
|  | C-Skeletal,Methoxy | 980 | 905 | Medium | High |
|  | C-O stretch | 1150 | 1070 | Variable | Mandatory |
|  | CH3 def, sym | 1450 | 1420 | Variable | Very high |
|  | CH3 def, asym | 1480 | 1435 | Variable | Very high |
|  | C-H stretch, Alkyl;Methoxy | 3000 | 2820 | Variable | Mandatory |
| 13 | 85 | Ethocy Silane | Silyl Ether | Aliphatic Silicon Compound |  |
|  | Corresponding Chemical | | | | |
|  | **Vibration** | **Start WN** | **End WN** | **Threshold** | **Priority** |
|  | Si-O stretch,sym | 990 | 945 | Variable | Mandatory |
|  | Si-O stretch,asym | 1100 | 1070 | Variable | Mandatory |
|  | Et-O rock | 1200 | 1145 | Variable | Mandatory |
|  | C-H stretch | 2885 | 2855 | Variable | Mandatory |
|  | C-H stretch | 2980 | 2950 | Variable | Very high |
| 14 | 81 | Aliphatic acid Halide, Long chain linear |  | Aliphatic acid halide |  |
|  | Corresponding chemical | | | | |
|  | **Vibration** | **Start WN** | **End WN** | **Threshold** | **Priority** |
|  | C-C=O,C-C stretch | 960 | 935 | Variable | Mandatory |
|  | C-C Skeletal | 1155 | 1120 | Variable | High |
|  | C-H Bend, Alpha CH2 | 1210 | 1190 | Variable | Mandatory |
|  | C-H Bend, CH2/CH3 | 1470 | 1445 | Variable | Mandatory |
|  | C=O stretch | 1825 | 1780 | Strong | Mandatory |
|  | C-H stretch, Alkyl | 2965 | 2915 | Variable | Medium |
| 15 | 81 | Isothiocyanate | Aliphatic Thiocyanate | Aliphatic Nitrile or Multiple bonded Nitrogen |  |
|  | Corresponding chemical | | | | |
|  | **Vibration** | **Start WN** | **End WN** | **Threshold** | **Priority** |
|  | N=C=S stretch,sym | 1240 | 1100 | Variable | Mandatory |
|  | C-H Bend,CH2/CH3 | 1470 | 1405 | Variable | Very high |
|  | N=C=S stretch, asym | 2145 | 2080 | Very strong | Mandatory |
|  | C-H stretch, Alkyl | 3000 | 2825 | Variable | Very high |
| 16 | 73 | CF3 group | Fluorine Compounds | Organic Halogen compounds |  |
|  | Corresponding chemical | | | | |
|  | **Vibration** | **Start WN** | **End WN** | **Threshold** | **Priority** |
|  | C-F Stretch | 1200 | 1105 | Variable | Mandatory |
|  | C-F stretch | 1280 | 1175 | Variable | Mandatory |
|  | C-F stretch | 1375 | 1260 | Variable | mandatory |

**Characterization of functional groups in control leaf ATR analysis**

| Functional Groups | | | | | |
| --- | --- | --- | --- | --- | --- |
| Sl NO | Quality | Functional Group | Chemical sub class | Chemical class |  |
| 1 | 100 | Aliphatic Cyanate | Aliphatic Cyanate | Aliphatic Nitrile or Multiple bonded Nitrogen |  |
|  | Corresponding chemical | | | | |
|  | **Vibration** | **Start WN** | **End WN** | **Threshold** | **Priority** |
|  | COCN Stretch | 1140 | 1080 | Variable | Mandatory |
|  | C-H Bend, CH2/CH3 | 1470 | 1435 | Variable | Mandatory |
|  | OCN stretch | 2265 | 2240 | Strong | Mandatory |
|  | C-H Stretch, Alkyl | 3000 | 2855 | Variable | High |
| 2 | 100 | Aliphatic Isonitrile | Aliphatic Nitrile | Aliphatic Nitrile or Multiple bonded Nitrogen |  |
|  | Corresponding chemical | | | | |
|  | **Vibration** | **Start WN** | **End WN** | **Threshold** | **Priority** |
|  | C-H Bend, CH2/CH3 | 1485 | 1445 | Variable | Mandatory |
|  | N≡C Stretch | 2170 | 2130 | Variable | Mandatory |
|  | C-H stretch,Alkyl | 3000 | 2855 | Variable | Medium |
| 3 | 100 | Carbonyl, Alpha Methylene | Substituent Group | Side chain or substituent |  |
|  | Corresponding chemical | | | | |
|  | **Vibration** | **Start WN** | **End WN** | **Threshold** | **Priority** |
|  | C-H bend, CH3 | 1395 | 1350 | Variable | Mandatory |
|  | C-H bend, Alpha CH2 | 1425 | 1395 | Variable | Mandatory |
|  | C-H bend, CH2/CH3 | 1475 | 1450 | Variable | Mandatory |
|  | C=O stretch | 1850 | 1690 | Strong | Mandatory |
|  | C-H stretch, Alkyl | 2970 | 2920 | Variable | Medium |
| 4 | 100 | Strained Ring or Activated Carbonyl | Substituent group | Side chain or substituent |  |
|  | Corresponding chemical | | | | |
|  | **Vibration** | **Start WN** | **End WN** | **Threshold** | **Priority** |
|  | C=O stretch | 1850 | 1750 | Strong | Mandatory |
|  | C-H stretch | 3090 | 2855 | Variable | Medium |
| 5 | 98 | Aliphatic acid or carbonyl compound |  | Aliphatic carboxylic acid |  |
|  | Corresponding chemical | | | | |
|  | **Vibration** | **Start WN** | **End WN** | **Threshold** | **Priority** |
|  | C-H bend, CH2/CH3 | 1450 | 1380 | Variable | Mandatory |
|  | C=O stretch | 1730 | 1680 | Strong | Mandatory |
|  | O-H Bonded, Acid | 2710 | 2580 | Variable | Mandatory |
|  | C-H stretch, Alkyl | 3120 | 2855 | Variable | Mandatory |
| 6 | 98 | Aliphatic Aldehyde |  | Aliphatic Aldehyde |  |
|  | Corresponding chemical | | | | |
|  | **Vibration** | **Start WN** | **End WN** | **Threshold** | **Priority** |
|  | C-H bend, CH2/CH3 | 1480 | 1410 | Variable | Mandatory |
|  | C=O stretch | 1740 | 1715 | Strong | Mandatory |
|  | C-H stretch, Aldehyde | 2765 | 2695 | Variable | Mandatory |
|  | C-H stretch, Alkyl | 3000 | 2855 | Variable | Mandatory |
| 7 | 98 | Aliphatic Alpha Amino acid |  | Aliphatic Amino acid |  |
|  | Corresponding chemical | | | | |
|  | **Vibration** | **Start WN** | **End WN** | **Threshold** | **Priority** |
|  | C-H bend | 1425 | 1390 | Variable | Mandatory |
|  | C-H bend, 1470 | 1470 | 1430 | Variable | Mandatory |
|  | N-H bend | 1530 | 1500 | Variable | Mandatory |
|  | C=O stretch | 1600 | 1560 | Strong | Mandatory |
|  | Bonded O-H | 2600 | 2560 | Variable | Mandatory |
|  | C-H stretch, Alkyl | 3000 | 2855 | Variable | Mandatory |
|  | N-H stretch, bonded | 3200 | 3050 | Variable | Mandatory |
| 8 | 98 | Aliphatic amido, Possibly subs. Urea |  | Aliphatic Amide |  |
|  | Corresponding chemical | | | | |
|  | **Vibration** | **Start WN** | **End WN** | **Threshold** | **Priority** |
|  | NCN stretch | 1195 | 1145 | Variable | Mandatory |
|  | NCN stretch | 1360 | 1310 | Variable | Mandatory |
|  | N-H bend, Amido | 1610 | 1530 | Strong | Mandatory |
|  | C=O Stretch,Amido | 1685 | 1630 | Strong | Mandatory |
|  | N-H stetch,Amido | 3445 | 3200 | Medium | Mandatory |
| 9 | 98 | Aliphatic Amino acid |  | Aliphatic Amino acid |  |
|  | Corresponding chemical | | | | |
|  | **Vibration** | **Start WN** | **End WN** | **Threshold** | **Priority** |
|  | C-H bend | 1425 | 1390 | Variable | Mandatory |
|  | C-H bend, CH2/CH3 | 1470 | 1430 | Variable | Mandatory |
|  | N-H bend | 1530 | 1500 | Variable | Mandatory |
|  | C=O stretch | 1600 | 1560 | Strong | Mandatory |
|  | Bonded O-H | 2600 | 2560 | Variable | Mandatory |
|  | C-H stretch, Alkyl | 3000 | 2855 | Variable | Mandatory |
| 10 | 98 | Aliphaic Amino acid – Carbonyl compound |  | Aliphatic Amino acid |  |
|  | Corresponding chemical | | | | |
|  | **Vibration** | **Start WN** | **End WN** | **Threshold** | **Priority** |
|  | C-H Bend, CH2/CH3 | 1450 | 1380 | Variable | Mandatory |
|  | C=O stretch | 1730 | 1690 | Variable | Mandatory |
|  | C-H stretch, Alkyl | 3000 | 2855 | Variable | Mandatory |
|  | N-H Stretch, Bonded | 3120 | 3060 | Variable | Mandatory |
| 11 | 98 | Aliphatic Carboxylate – Carbonyl compound |  | Aliphatic Carboxylate |  |
|  | Corresponding chemical | | | | |
|  | **Vibration** | **Start WN** | **End WN** | **Threshold** | **Priority** |
|  | C-H bend, CH3 | 1380 | 1360 | Weak | Mandatory |
|  | C-O stretch, Carboxylate | 1445 | 1400 | Medium | Mandatory |
|  | C-H bend, CH2/CH3 | 1470 | 1450 | Weak | Mandatory |
|  | C-O stretch, Carboxylate | 1605 | 1540 | Strong | Mandatory |
|  | C-H stretch, Alkyl | 2975 | 2855 | Variable | Medium |
| 12 | 98 | Aromatic amino acid, Bonded |  | Aromatic amino aid |  |
|  | Corresponding chemical | | | | |
|  | **Vibration** | **Start WN** | **End WN** | **Threshold** | **Priority** |
|  | C-H bend, OOP | 840 | 635 | Variable | Mandatory |
|  | C-N stretch | 1415 | 1385 | Variable | Mandatory |
|  | C-H bend | 1465 | 1430 | Variable | Mandatory |
|  | C-C Stretch, Ring | 1515 | 1445 | Strong | Mandatory |
|  | N-H bend | 1600 | 1555 | Medium | Mandatory |
|  | C=O stretch | 1655 | 1600 | Variable | Mandatory |
|  | O-HH- bonded | 2650 | 2500 | Variable | Mandatory |
|  | C-H stretch/N-H bonded | 3070 | 2930 | Variable | Mandatory |
| 13 | 98 | Aromatic amino acid, Free amino |  | Aromatic amino acid |  |
|  | Corresponding chemical | | | | |
|  | **Vibration** | **Start WN** | **End WN** | **Threshold** | **Priority** |
|  | C-H bend, OOP | 840 | 635 | Variable | Mandatory |
|  | C-N stretch | 1365 | 1305 | Variable | Mandatory |
|  | C-H bend | 1465 | 1430 | Variable | Mandatory |
|  | N-H bend | 1560 | 1535 | Medium | Mandatory |
|  | C=O stretch | 1665 | 1620 | Variable | Mandatory |
|  | C=O stretch | 1730 | 1695 | Variable | Mandatory |
|  | C-H stretch | 3090 | 2855 | Variable | Medium |
|  | N-H stretch | 3400 | 3300 | Variable | Mandatory |
| 14 | 98 | Carbonyl, Possibly 2-Hydroxy acid |  | Aliphatic Carboxy acid |  |
|  | Corresponding chemical | | | | |
|  | **Vibration** | **Start WN** | **End WN** | **Threshold** | **Priority** |
|  | O-H Bonded | 950 | 880 | Variable | Mandatory |
|  | C-H Bend, CH2/CH3 | 1475 | 1400 | Variable | Mandatory |
|  | C=O stretch, Conjugated | 1720 | 1680 | Strong | Mandatory |
|  | O-H bonded,Acid | 2710 | 2580 | Variable | Mandatory |
|  | C-H stretch, Alkyl | 2990 | 2900 | Variable | Mandatory |
|  | O-H stretch, 2-Hydroxy | 3500 | 3380 | Variable | Mandatory |
| 15 | 98 | Carbonyl, Possibly Conjugated Acid |  | Aliphatic Carboxylic Acid |  |
|  | Corresponding chemical | | | | |
|  | **Vibration** | **Start WN** | **End WN** | **Threshold** | **Priority** |
|  | O-H bonded/CH bend | 960 | 905 | Variable | Mandatory |
|  | C-H bend, CH2/CH3 | 1475 | 1400 | Variable | Mandatory |
|  | C=C stretch, Conjugated | 1655 | 1620 | Variable | Mandatory |
|  | C=O stretch, Conjugated | 1705 | 1680 | Strong | Mandatory |
|  | O-H Bonded, Acid | 2710 | 2580 | Variable | Mandatory |
|  | C-H stretch, Alkyl | 2990 | 2900 | Variable | Mandatory |
| 16 | 98 | Carboxylic acid, General |  | Aliphatic carboxylic acid |  |
|  | Corresponding chemical | | | | |
|  | **Vibration** | **Start WN** | **End WN** | **Threshold** | **Priority** |
|  | O-H bonded,OOP | 945 | 910 | Variable | Mandatory |
|  | C-H bend, CH2/CH3 | 1450 | 1380 | Variable | Mandatory |
|  | C=O stretch | 1730 | 1680 | Strong | Mandatory |
|  | O-H bonded, Acid | 2710 | 2580 | Variable | Mandatory |
|  | C-H stretch, Alkyl | 3120 | 2855 | Variable | Mandatory |
| 17 | 96 | Aliphatic Amine, Primary, Branched |  | Aliphatic Amine |  |
|  | Corresponding chemical | | | | |
|  | **Vibration** | **Start WN** | **End WN** | **Threshold** | **Priority** |
|  | N-H Deformation | 850 | 760 | Variable | Mandatory |
|  | C-N stretch | 1140 | 1080 | Variable | Mandatory |
|  | C-H Def, CH | 1350 | 1320 | Variable | High |
|  | C-H bend, CH3 | 1395 | 1370 | Variable | High |
|  | C-H bend, CH2/CH3 | 1480 | 1455 | Variable | High |
|  | N-H bend | 1625 | 1590 | Variable | Mandatory |
|  | C-H stretch, CH2 | 2970 | 2895 | Variable | Mandatory |
|  | N-H stretch | 3420 | 3270 | Variable | Mandatory |
|  | N-H stretch | 3550 | 3445 | Variable | Mandatory |
| 18 | 96 | N-Methylamino Substituent | Substituent Group | Side chain or Substituent |  |
|  | Corresponding chemical | | | | |
|  | **Vibration** | **Start WN** | **End WN** | **Threshold** | **Priority** |
|  | C-H bend, CH3 | 1380 | 1360 | Variable | Mandatory |
|  | C-H bend, CH/CH3 | 1470 | 1450 | Variable | Mandatory |
|  | C-H stretch, CH3 | 2800 | 2750 | Variable | Mandatory |
|  | C-H stretch, CH2 | 2980 | 2920 | Variable | Medium |
| 19 | 92 | Aliphatic conjugated Ester |  | Aliphatic ester |  |
|  | Corresponding chemical | | | | |
|  | **Vibration** | **Start WN** | **End WN** | **Threshold** | **Priority** |
|  | COC def | 840 | 770 | Medium | High |
|  | COC stretch, sym | 1190 | 1130 | Variable | Mandatory |
|  | COC stretch, asym | 1315 | 1260 | Variable | Mandatory |
|  | C-H Bend, CH2/CH3 | 1480 | 1410 | Variable | Very high |
|  | C=C stretch | 1645 | 1580 | Variable | Mandatory |
|  | C=O stretch | 1740 | 1700 | Variable | Mandatory |
|  | C-H stretch, Alkyl | 3000 | 2890 | Variable | Very high |
|  | C-H stretch, olefinic | 3040 | 3010 | Variable | Mandatory |
| 20 | 91 | Furan, 2 susbst. | Furans | Other Aromatic compounds |  |
|  | Corresponding chemical | | | | |
|  | **Vibration** | **Start WN** | **End WN** | **Threshold** | **Priority** |
|  | C-H def | 1180 | 1140 | Variable | Mandatory |
|  | C-H def | 1250 | 1200 | Variable | Mandatory |
|  | C=C stretch | 1410 | 1385 | Variable | High |
|  | C=C stretch | 1520 | 1460 | Variable | Mandatory |
|  | C=C stretch | 1610 | 1560 | Variable | Mandatory |
|  | C-H stretch, arom | 3180 | 3000 | Medium | Mandatory |
| 21 | 90 | Aliphatic Alkoxy, Methoxy |  | Aliphatic Ether |  |
|  | Corresponding chemical | | | | |
|  | **Vibration** | **Start WN** | **End WN** | **Threshold** | **Priority** |
|  | C Skeletal, Methoxy | 980 | 905 | Medium | High |
|  | C-O stretch | 1150 | 1070 | Variable | Mandatory |
|  | CH3 def, asym | 1480 | 1435 | Variable | Very high |
|  | CH3 def, sym | 1450 | 1420 | Variable | Very high |
|  | C-H stretch, Alkyl;Methoxy | 3000 | 2820 | Variable | Mandatory |
| 22 | 90 | Pyridines, General | Pyridines | Other aromatic compounds |  |
|  | Corresponding chemical | | | | |
|  | **Vibration** | **Start WN** | **End WN** | **Threshold** | **Priority** |
|  | C-H in plane def | 1300 | 1200 | Variable | High |
|  | C=C and S=N stretch | 1450 | 1410 | Variable | Mandatory |
|  | C=C and C=N stretch | 1520 | 1470 | Variable | Mandatory |
|  | C=C and C=N stretch | 1615 | 1570 | Variable | Mandatory |
|  | C-H stretch | 3100 | 3010 | Variable | Mandatory |
| 23 | 90 | Pyrroles, 2 subst. | Pyrroles | Other Aromatic compounds |  |
|  | Corresponding chemical | | | | |
|  | **Vibration** | **Start WN** | **End WN** | **Threshold** | **Priority** |
|  | C-H def | 900 | 870 | Medium | High |
|  | C=C and C=N stretch | 1515 | 1460 | Medium | Mandatory |
|  | C=C and C=N stretch | 1605 | 1560 | Medium | Mandatory |
|  | N-H stretch | 3400 | 3000 | Strong | Very High |
|  | C-H stretch, arom | 3100 | 3010 | Medium | Mandatory |
| 24 | 85 | Aliphatic Primary Amide |  | Aliphatic Amide |  |
|  | Corresponding chemical | | | | |
|  | **Vibration** | **Start WN** | **End WN** | **Threshold** | **Priority** |
|  | C-N stretch | 1420 | 1400 | Variable | Very high |
|  | C-H bend | 1480 | 1425 | Medium | High |
|  | N-H def | 1650 | 1620 | Variable | Mandatory |
|  | C=O stretch | 1675 | 1645 | Variable | Mandatory |
|  | C-H stretch, Alkyl | 2990 | 2850 | Variable | High |
|  | N-H stretch | 3220 | 3150 | Variable | Mandatory |
|  | N-H stretch, Amido | 3400 | 3300 | Variable | Mandatory |
| 25 | 84 | Unsaturated Hydrocarbon, Cyclic,> C5 |  | Alkene |  |
|  | Corresponding chemical | | | | |
|  | **Vibration** | **Start WN** | **End WN** | **Threshold** | **Priority** |
|  | C-H bend, Alkyl | 1470 | 1430 | Variable | High |
|  | C=C stretch, Alkene | 1650 | 1605 | Variable | Mandatory |
|  | C-H stretch, Alkyl | 3000 | 2900 | Variable | High |
|  | C-H stretch, Alkene | 3090 | 3005 | Variable | High |
| 26 | 83 | 4-Subst Pyridine | Pyridines | Other Aromatic compounds |  |
|  | Corresponding chemical | | | | |
|  | **Vibration** | **Start WN** | **End WN** | **Threshold** | **Priority** |
|  | C=C and C=N stretch | 1420 | 1400 | Variable | Mandatory |
|  | C=C and C=N stretch | 1520 | 1480 | Medium | Mandatory |
|  | C=C and C=N stretch | 1570 | 1550 | Medium | Mandatory |
|  | C-H stretch | 3100 | 3010 | Variable | Mandatory |
|  | C=C and C=N stretch | 1610 | 1565 | Variable | Mandatory |
| 27 | 82 | Unsaturated Hydrocarbon, Vinylidene |  | Alkene |  |
|  | Corresponding chemical | | | | |
|  | **Vibration** | **Start WN** | **End WN** | **Threshold** | **Priority** |
|  | C-H bend, Alkene, OOP | 895 | 865 | Variable | Mandatory |
|  | C-H bend, Alkyl | 1480 | 1440 | Variable | High |
|  | C=C stretch, Alkene | 1665 | 1635 | Variable | Mandatory |
|  | C-H stretch, Alkyl | 3000 | 2900 | Variable | High |
|  | C-H stretch, Alkene | 3095 | 3050 | Variable | Mandatory |
| 28 | 81 | Unsaturated Hydrocarbon, Trans Conjugated |  | Alkene |  |
|  | Corresponding chemical | | | | |
|  | **Vibration** | **Start WN** | **End WN** | **Threshold** | **Priority** |
|  | C-H Bend, Alkyl | 1470 | 1430 | Variable | Very high |
|  | C=C stretch, Alkene | 1660 | 1585 | Variable | Mandatory |
|  | C-H stretch, Alkyl | 3000 | 2900 | Variable | Very high |
|  | C-H stretch, Alkene | 3050 | 3010 | Variable | Mandatory |
| 29 | 79 | 2-Subst Pyridine | Pyridines | Other Aromatic compounds |  |
|  | Corresponding group | | | | |
|  | **Vibration** | **Start WN** | **End WN** | **Threshold** | **Priority** |
|  | C=C and C=N stretch | 1440 | 1420 | Variable1 | Mandatory |
|  | C=C and C=N stretch | 1480 | 1450 | Variable | Mandatory |
|  | C=C and C=N stretch | 1615 | 1570 | Variable | Mandatory |
|  | C-H stretch | 3100 | 3010 | Variable | Mandatory |
| 30 | 79 | Aliphatic Tertiary |  | Aliphatic amide |  |
|  | Corresponding chemical | | | | |
|  | **Vibration** | **Start WN** | **End WN** | **Threshold** | **Priority** |
|  | C-N stretch/C-C stretch | 1150 | 1050 | Variable | Mandatory |
|  | C-H bend | 1460 | 1410 | Variable | Mandatory |
|  | C=O stretch, Amido | 1690 | 1645 | Strong | Mandatory |
|  | C-H stretch, Alkyl | 3000 | 2855 | Variable | Medium |
| 31 | 78 | Aliphatic Ester, Acetate |  | Aliphatic Ester |  |
|  | Corresponding chemical | | | | |
|  | **Vibration** | **Start WN** | **End WN** | **Threshold** | **Priority** |
|  | C-O-C stretch | 1265 | 1205 | Variable | Mandatory |
|  | C-H bend, CH2/CH3 | 1475 | 1425 | Variable | High |
|  | C=O stretch | 1755 | 1730 | Variable | Mandatory |
|  | C-H stretch, Alkyl | 3000 | 2855 | Variable | High |
| 32 | 76 | Unsaturated Hydrocarbon, Trisubstituted |  | Alkene |  |
|  | Corresponding chemical | | | | |
|  | **Vibration** | **Start WN** | **End WN** | **Threshold** | **Priority** |
|  | C-H bend, Alkene,OOP | 855 | 785 | Weak | High |
|  | C-H bend, Alkyl | 1480 | 1435 | Variable | Mandatory |
|  | C=C stretch, Alkene | 1695 | 1665 | Variable | Mandatory |
|  | C-H stretch, Alkyl | 3000 | 2900 | Variable | High |
|  | C-H stretch, Alkene | 3045 | 3005 | Variable | Mandatory |
| 33 | 72 | CF3 Group | Fluorine Compounds | Organic Halogen Compounds |  |
|  | Corresponding chemical | | | | |
|  | **Vibration** | **Start WN** | **End WN** | **Threshold** | **Priority** |
|  | C-F stretch | 1200 | 1105 | Variable | Mandatory |
|  | C-F stretch | 1280 | 1175 | Variable | Mandatory |
|  | C-F stretch | 1375 | 1260 | Variable | Mandatory |
| 34 | 69 | Unsaurated Hydrocarbon, Ether Conjugated |  | Alkene |  |
|  | Corresponding chemical | | | | |
|  | **Vibration** | **Start WN** | **End WN** | **Threshold** | **Priority** |
|  | C-O Stretch, Ether | 850 | 810 | Weak | Medium |
|  | C-O stretch, Ether | 1240 | 1200 | Variable | Mandatory |
|  | C=C stretch, Alkene | 1650 | 1610 | Variable | Mandatory |
|  | C-H stretch, Alkyl | 2975 | 2900 | Variable | Mandatory |

**Characterization of functional groups in third Stage of Gall around ATR analysis**

| Functional Groups | | | | | |
| --- | --- | --- | --- | --- | --- |
| Sl NO | Quality | Functional Groups | Chemical sub class | Chemical class |  |
| 1 | 100 | Aliphatic Anhydride – carbonyl compound |  | Aliphatic anhydride |  |
|  | Corresponding chemical | | | | |
|  | **Vibration** | **Start WN** | **End WN** | **Threshold** | **Priority** |
|  | C-O Stretch | 1075 | 1005 | Variable | Mandatory |
|  | C-H Bend, CH2/CH3 | 1470 | 1425 | Variable | Very high |
|  | C=O stretch | 1785 | 1740 | Variable | Mandatory |
|  | C=O stretch | 1850 | 1805 | Variable | Mandatory |
|  | C-H stretch, Alkyl | 3000 | 2855 | Variable | High |
| 2 | 100 | Cyclic Anhydride, Five membered |  | Aliphatic Anhydride |  |
|  | Corresponding chemical | | | | |
|  | **Vibration** | **Start WN** | **End WN** | **Threshold** | **Priority** |
|  | C-O-C stretch | 950 | 900 | Strong | Mandatory |
|  | C-O-C stretch | 1300 | 1220 | Variable | Mandatory |
|  | C-H bend, Alpha CH | 1420 | 1400 | Variable | High |
|  | C=O stretch | 1805 | 1770 | Variable | Mandatory |
|  | C=O stretch | 1875 | 1825 | Variable | Mandatory |
|  | C-H stretch, Alkyl | 3000 | 2855 | Variable | Medium |
| 3 | 100 | Metal carbonyl, Terminal + Aryl |  | Metal carbonyl |  |
|  | Corresponding chemical | | | | |
|  | **Vibration** | **Start WN** | **End WN** | **Threshold** | **Priority** |
|  | Ring, C-H bend | 640 | 625 | Variable | High |
|  | Ring, C-H bend | 685 | 655 | Variable | Mandatory |
|  | C=O Metal carbonyl | 1890 | 1840 | Very strong | Mandatory |
|  | C=O Metal carbonyl | 1990 | 1920 | Very strong | Mandatory |
| 4 | 100 | Metal carbonyl, Terminal + Cyclopentadienyl |  | Metal Carbonyl |  |
|  | Corresponding chemical | | | | |
|  | **Vibration** | **Start WN** | **End WN** | **Threshold** | **Priority** |
|  | Ring, C-H bend | 845 | 810 | Variable | Mandatory |
|  | C=O Metal carbonyl | 1985 | 1890 | Very strong | Mandatory |
|  | C=O Metal carbonyl | 2100 | 1930 | Very strong | Mandatory |
|  | C-H stretch, Alkyl | 2975 | 2855 | Variable | Mandatory |
| 5 | 100 | Metal carbonyl, Terminal or Metal Cyano complex |  | Metal carbonyl |  |
|  | Corresponding chemical | | | | |
|  | **Vibration** | **Start WN** | **End WN** | **Threshold** | **Priority** |
|  | C=O Metal Carbonyl | 2100 | 1900 | Very strong | Mandatory |
| 6 | 99 | Aliphatic carbodiimide | Aliphatic carbodiimide | Aliphatic Nitrile or Multiple bonded Nitrogen |  |
|  | Corresponding chemical | | | | |
|  | **Vibration** | **Start WN** | **End WN** | **Threshold** | **Priority** |
|  | C-H bend, CH2/CH3 | 1470 | 1405 | Variable | Very high |
|  | N=C=N stretch, sym | 1500 | 1440 | Variable | Mandatory |
|  | N=C=N stretch, asym | 2160 | 2130 | Very strong | Mandatory |
|  | C-H stretch, Alkyl | 3000 | 2825 | Variable | Very high |
| 7 | 99 | Aliphatic Isocyanate | Aliphatic cyanate | Aliphatic Nitrile or multiple bonded nitrogen |  |
|  | Corresponding chemical | | | | |
|  | **Vibration** | **Start WN** | **End WN** | **Threshold** | **Priority** |
|  | NCO Def | 645 | 590 | Variable | Mandatory |
|  | C-N bend | 890 | 830 | Variable | Mandatory |
|  | NCO stretch, sym | 1440 | 1360 | Variable | Mandatory |
|  | C-H bend, CH2/CH3 | 1470 | 1445 | Variable | Mandatory |
|  | NCO stretch, asym | 2295 | 2250 | Very strong | Mandatory |
|  | C-H stretch, Alkyl | 3000 | 2825 | Variable | Medium |
| 8 | 99 | Aliphatic Isonitrile | Aliphatic Nitrile | Aliphatic nitrile or multiple bonded nitrogen |  |
|  | Corresponding chemical | | | | |
|  | **Vibration** | **Start WN** | **End WN** | **Threshold** | **Priority** |
|  | C-H bend, CH2/CH3 | 1485 | 1445 | Variable | Mandatory |
|  | N≡C Stretch | 2170 | 2130 | Variable | Mandatory |
|  | C-H stretch, Alkyl | 3000 | 2855 | Variable | Medium |
| 9 | 99 | Aliphatic Nitrile | Aliphatic Nitrile | Aliphatic nitrile or multiple bonded nitrogen |  |
|  | Corresponding chemical | | | | |
|  | **Vibration** | **Start WN** | **End WN** | **Threshold** | **Priority** |
|  | CCN in Plane | 580 | 530 | Variable | Mandatory |
|  | C-H bend, CH2/CH3 | 1470 | 1425 | Variable | Mandatory |
|  | C≡N Stretch, Nitrile | 2260 | 2230 | Variable | Mandatory |
|  | C-H stretch, Alkyl | 3000 | 2855 | Variable | Medium |
| 10 | 99 | Aliphatic nitrile, Conjugated | Aliphatic Nitrile | Aliphatic nitrile or multiple bonded nitrogen |  |
|  | Corresponding chemical | | | | |
|  | **Vibration** | **Start WN** | **End WN** | **Threshold** | **Priority** |
|  | C-H bend, OOP | 980 | 925 | Variable | Mandatory |
|  | C=C stretch | 1660 | 1620 | Variable | Mandatory |
|  | C≡N Stretch, Nitrile | 2250 | 2200 | Variable | Mandatory |
|  | C-H stretch, olefin | 3095 | 3000 | Variable | Medium |
| 11 | 99 | Aliphatic Thiocyanate | Aliphatic Thiocyanate | Aliphatic nitrile or multiple bonded Nitrogen |  |
|  | Corresponding chemical | | | | |
|  | **Vibration** | **Start WN** | **End WN** | **Threshold** | **Priority** |
|  | SCN bend | 510 | 460 | Variable | High |
|  | C-S-C | 660 | 620 | Variable | Mandatory |
|  | C-S-C | 700 | 680 | Weak | Very high |
|  | S-C≡N sym Stretch | 1050 | 950 | Weak | Very high |
|  | C-H bend, CH2/CH3 | 1470 | 1425 | Variable | Mandatory |
|  | S-C≡N asym Stretch | 2165 | 2140 | Variable | Mandatory |
|  | C-H stretch, Alkyl | 3000 | 2855 | Variable | Medium |
| 12 | 99 | Isothiocyanate | Aliphatic thiocyanate | Aliphatic Nitrile or multiple bonded nitrogen |  |
|  | Corresponding chemical | | | | |
|  | **Vibration** | **Start WN** | **End EN** | **Threshold** | **Priority** |
|  | NCS OOP | 560 | 520 | Variable | Very high |
|  | NCS def | 640 | 600 | Variable | High |
|  | N=C=S stretch, sym | 1240 | 1100 | Variable | Mandatory |
|  | C-H bend, CH2/CH3 | 1470 | 1405 | Variable | Very high |
|  | N=C=S stretch, asym | 2145 | 2080 | Very strong | Mandatory |
|  | C-H stretch, Alkyl | 3000 | 2825 | Variable | Very high |
| 13 | 94 | Aromatic amino acid, Bonded |  | Aromatic amino acid |  |
|  | Corresponding chemical | | | | |
|  | **Vibration** | **Start WN** | **End WN** | **Threshold** | **Priority** |
|  | C-H bend, OOP | 840 | 635 | Variable | Mandatory |
|  | C-N stretch | 1415 | 1385 | Variable | Mandatory |
|  | C-H bend | 1465 | 1430 | Variable | Mandatory |
|  | C-C stretch, Ring | 1515 | 1445 | Strong | Mandatory |
|  | N-H bend | 1600 | 1555 | Medium | Mandatory |
|  | C=O stretch | 1655 | 1600 | Variable | Mandatory |
|  | O-H H -bonded | 2650 | 2500 | Variable | Mandatory |
|  | C-H stretch/N-H bonded | 3070 | 2930 | Variable | Mandatory |
| 14 | 94 | Tetramethyl Ammonium salt |  | Aliphatic Amine |  |
|  | Corresponding chemical | | | | |
|  | **Vibration** | **Start WN** | **End WN** | **Threshold** | **Priority** |
|  | C-N stretch | 955 | 940 | Variable | Mandatory |
|  | C-H bend, CH3 | 1420 | 1395 | Variable | Mandatory |
|  | C-H bend, CH3 | 1500 | 1480 | Variable | Mandatory |
|  | C-H stretch, CH3 | 3030 | 2985 | Variable | Mandatory |
| 15 | 92 | Aliphatic Alkoxy, Methoxy |  | Aliphatic Ether |  |
|  | Corresponding chemical | | | | |
|  | **Vibration** | **Start WN** | **End WN** | **Threshold** | **Priority** |
|  | CO def | 560 | 400 | Weak | Medium |
|  | S Skeletal, Methoxy | 980 | 905 | Medium | High |
|  | C-O stretch | 1150 | 1070 | Variable | Mandatory |
|  | CH3 def, asym | 1480 | 1435 | Variable | Very high |
|  | C-H stretch, Alkyl; Methoxy | 3000 | 2820 | Variable | Mandatory |
| 16 | 92 | Aliphatic conjugated Carboxylic acid | Conjugated carboxylic acid | Aromatic or Conjugated carboxylic acid |  |
|  | Corresponding chemical | | | | |
|  | **Vibration** | **Start WN** | **End WN** | **Threshold** | **Priority** |
|  | C-O-H Def | 950 | 880 | Variable | Mandatory |
|  | C-O stretch | 1320 | 1260 | Strong | Mandatory |
|  | C=O stretch | 1650 | 1610 | Variable | Mandatory |
|  | C=O stretch | 1700 | 1665 | Strong | Mandatory |
|  | O-H stretch, Dimeric | 2680 | 2580 | Variable | Mandatory |
|  | C-H stretch, Alkyl | 3000 | 2855 | Variable | Mandatory |
| 17 | 92 | Aliphatic sulfonate or sulfate | Aliphatic sulfoxy- compound | Aliphatic sulfur compound |  |
|  | Corresponding chemical | | | | |
|  | **Vibration** | **Start WN** | **End WN** | **Threshold** | **Priority** |
|  | S-O stretch, sym | 830 | 700 | Variable | Very high |
|  | S-O stretch | 1010 | 900 | Strong | Mandatory |
|  | S=O stretch, sym | 1210 | 1150 | Strong | Mandatory |
|  | S=O stretch, asym | 1420 | 1340 | Strong | Mandatory |
|  | C-H bend, CH2/CH3 | 1480 | 1410 | Variable | Mandatory |
|  | C-H stretch, Alkyl | 3000 | 2855 | Variable | High |
| 18 | 92 | Aliphatic tert-Amino acid |  | Aliphatic amino acid |  |
|  | Corresponding chemical | | | | |
|  | **Vibration** | **start WN** | **End WN** | **Threshold** | **Priority** |
|  | N-H bend | 750 | 690 | Variable | Mandatory |
|  | C-H bend | 1425 | 1390 | Variable | Mandatory |
|  | C-H bend, CH2/CH3 | 1470 | 1430 | Variable | Mandatory |
|  | N-H bend | 1530 | 1500 | Variable | Mandatory |
|  | C=O stretch | 1740 | 1640 | Strong | Mandatory |
|  | C-H stretch, Alkyl | 3000 | 2855 | Variable | Mandatory |
|  | N-H stretch, Bonded | 3300 | 3100 | Variable | Mandatory |
| 19 | 92 | Carbonyl, Possibly 2-Hydroxy acid |  | Aliphatic carboxylic acid |  |
|  | Corresponding chemical | | | | |
|  | **Vibration** | **Start WN** | **End WN** | **Threshold** | **Priority** |
|  | O-H bonded | 950 | 880 | Variable | Mandatory |
|  | C-H bend, CH2/CH3 | 1475 | 1400 | Variable | Mandatory |
|  | C=O stretch, conjugated | 1720 | 1680 | Strong | Mandatory |
|  | O-H bonded, Acid | 2710 | 2580 | Variable | Mandatory |
|  | C-H stretch, Alkyl | 2990 | 2900 | Variable | Mandatory |
|  | O-H stretch, 2-Hydroxy | 3500 | 3380 | Variable | Mandatory |
| 20 | 92 | Carbonyl, possibly conjugated acid |  | Aliphatic carboxylic acid |  |
|  | Corresponding chemical | | | | |
|  | **Vibration** | **Start WN** | **End WN** | **Threshold** | **Priority** |
|  | O-H bonded/CH bend | 960 | 905 | Variable | Mandatory |
|  | C-H bend, CH2/CH3 | 1475 | 1400 | Variable | Mandatory |
|  | C=C stretch, conjugated | 1655 | 1620 | Variable | Mandatory |
|  | C=O stretch, conjugated | 1705 | 1680 | Strong | Mandatory |
|  | O-H bonded, Acid | 2710 | 2580 | Variable | Mandatory |
|  | C-H stretch, Alkyl | 2990 | 2900 | Variable | Mandatory |
| 21 | 92 | Carboxylic acid, General |  | Aliphatic carboxylic acid |  |
|  | Corresponding chemical | | | | |
|  | **Vibration** | **Start WN** | **End WN** | **Threshold** | **Priority** |
|  | O-H bonded, OOP | 945 | 910 | Variable | Mandatory |
|  | C-H bend, CH2/CH3 | 1450 | 1380 | Variable | Mandatory |
|  | C=O stretch | 1730 | 1680 | Strong | Mandatory |
|  | O-H bonded, Acid | 2710 | 2580 | Variable | Mandatory |
|  | C-H stretch, Alkyl | 3120 | 2855 | Variable | Mandatory |
| 22 | 92 | Carboxylic acid, Linear chain |  | Aliphatic carboxylic acid |  |
|  | Corresponding chemiacl | | | | |
|  | **Vibration** | **Start WN** | **End WN** | **Threshold** | **Priority** |
|  | O-H bonded, OOP | 945 | 910 | Variable | Mandatory |
|  | C-O stretch, Acid | 1245 | 1225 | Variable | Mandatory |
|  | C-O stretch, Acid | 1300 | 1275 | Variable | Mandatory |
|  | C-H bend, Alpha CH2 | 1420 | 1390 | Variable | Mandatory |
|  | C-H bend, CH2/CH3 | 1475 | 1455 | Variable | Mandatory |
|  | C=O stretch | 1720 | 1680 | Strong | Mandatory |
|  | O-H bonded, Acid | 2710 | 2580 | Variable | Mandatory |
|  | C-H stretch, Alkyl | 2990 | 2900 | Variable | Mandatory |
| 23 | 92 | Possibly Thiophene derivative | Aryl group | Aromatic compound |  |
|  | Corresponding chemical | | | | |
|  | **Vibration** | **Start WN** | **End WN** | **Threshold** | **Priority** |
|  | C-H OOP bend | 800 | 760 | Strong | Mandatory |
|  | C-H OOP bend | 860 | 835 | Strong | Mandatory |
|  | C-C/C-S stretch, Ring | 1445 | 1395 | Variable | Mandatory |
|  | C-C/C-S stretch, Ring | 1565 | 1515 | Variable | Mandatory |
|  | C-H stretch, Aryl | 3115 | 3050 | Variable | Medium |
| 24 | 92 | Unsaturated Hydrocarbon, Generic, Isolated |  | Alkene |  |
|  | Corresponding chemical | | | | |
|  | **Vibration** | **Start WN** | **End WN** | **Threshold** | **Priority** |
|  | C-H bend, Alkene, OOP | 930 | 875 | Strong | Mandatory |
|  | C-H bend, Alkyl | 1480 | 1435 | Variable | Mandatory |
|  | C=C stretch, isolated | 1680 | 1620 | Variable | Mandatory |
|  | C-H stretch, alkyl | 3000 | 2900 | Variable | Mandatory |
|  | C-H stretch, Alkene | 3050 | 3005 | Variable | Medium |
| 25 | 90 | Aliphatic acid or Carbonyl compound |  | Aliphatic carboxylic acid |  |
|  | Corresponding chemical | | | | |
|  | **Vibration** | **Start WN** | **End WN** | **Threshold** | **Priority** |
|  | C-H bend, CH2/CH3 | 1450 | 1380 | Variable | Mandatory |
|  | C=O stretch | 1730 | 1680 | Strong | Mandatory |
|  | O-H bonded, Acid | 2710 | 2580 | Variable | Mandatory |
|  | C-H stretch, Alkyl | 3120 | 2855 | Variable | Mandatory |
| 26 | 90 | Aliphatic amino acid – carbonyl compound |  | Aliphatic amino acid |  |
|  | Corresponding chemical | | | | |
|  | **Vibration** | **Start WN** | **End WN** | **Threshold** | **Priority** |
|  | C-H bend, CH2/CH3 | 1450 | 1380 | Variable | Mandatory |
|  | C=O stretch | 1730 | 1690 | Variable | Mandatory |
|  | C-H stretch, Alkyl | 3000 | 2855 | Variable | Mandatory |
|  | N-H stretch, bonded | 3120 | 3060 | Variable | Mandatory |
| 27 | 90 | Aliphatic ether or Sulfone | Substituent group | Side chain or substituent |  |
|  | Corresponding chemical | | | | |
|  | **Vibration** | **Start WN** | **End WN** | **Threshold** | **Priority** |
|  | SO2, Sulfoxy stretch | 1150 | 1115 | Strong | Mandatory |
|  | SO2, Sulfoxy stretch | 1325 | 1275 | Strong | Mandatory |
|  | C-H bend, CH2 | 1470 | 1415 | Variable | Mandatory |
|  | C-H stretch, Alkyl | 3000 | 2855 | Variable | Mandatory |
| 28 | 90 | CF3 group | Fluorine compounds | Organic Halogen compound |  |
|  | Corresponding chemical | | | | |
|  | **Vibration** | **Start WN** | **End WN** | **Threshold** | **Priority** |
|  | C-F def | 620 | 510 | Weak | High |
|  | C-F def | 770 | 650 | Weak | Very high |
|  | C-F stretch | 1200 | 1105 | Variable | Mandatory |
|  | C-F stretch | 1280 | 1175 | Variable | Mandatory |
|  | C-F stretch | 1375 | 1260 | Variable | Mandatory |
| 29 | 90 | Methyl Mercapto | Aliphatic Thioether | Aliphatic Thiocompounds |  |
|  | Corresponding chemical | | | | |
|  | **Vibration** | **Start WN** | **End WN** | **Threshold** | **Priority** |
|  | C-S stretch | 750 | 685 | Variable | Mandatory |
|  | Methyl CH3-S- | 1000 | 960 | Variable | Mandatory |
|  | C-H bend, CH3 | 1340 | 1300 | Variable | Very high |
|  | C-H bend, CH3 | 1455 | 1405 | Variable | Very high |
|  | C-H stretch, CH3-S- | 3030 | 2855 | Variable | Very high |
| 30 | 89 | Aliphatic carboxylate, Possibly trifluoroacetate |  | Aliphatic carboxylate |  |
|  | Corresponding chemical | | | | |
|  | **Vibration** | **Start WN** | **End WN** | **Threshold** | **Priority** |
|  | C-O bend, Carboxylate | 735 | 715 | Variable | Mandatory |
|  | C-O bend, carboxylate | 805 | 775 | Variable | Mandatory |
|  | Skeletal | 860 | 835 | Variable | Mandatory |
|  | C-F stretch | 1215 | 1170 | Strong | Mandatory |
|  | C-O stretch, carboxylate | 1465 | 1440 | Medium | Mandatory |
|  | C-O stretch, carboxylate | 1695 | 1650 | Strong | Mandatory |
| 31 | 89 | Aliphatic ether or sulfonate salt | Substituent group | Side chain or substituent |  |
|  | Corresponding chemical | | | | |
|  | **Vibration** | **Start WN** | **End WN** | **Threshold** | **Priority** |
|  | SO2, Sulfoxy stretch | 1065 | 1030 | Variable | Mandatory |
|  | SO2, Sulfoxy stretch | 1205 | 1160 | Strong | Mandatory |
|  | C-H bend, CH2 | 1470 | 1415 | Variable | Mandatory |
|  | C-H stretch, Alkyl | 3000 | 2855 | Variable | Mandatory |
| 32 | 89 | Aliphatic Hydrated sulfonate salt or Alcohol | Substituent group | Side chain or substituent |  |
|  | Corresponding chemical | | | | |
|  | **Vibration** | **Start WN** | **End WN** | **Threshold** | **Priority** |
|  | C-S stretch | 680 | 645 | Variable | Mandatory |
|  | C-S, CH2 bend | 1290 | 1220 | Variable | Mandatory |
|  | C-H bend, CH2/CH3 | 1480 | 1410 | Variable | Mandatory |
|  | S-H stretch | 2565 | 2525 | Variable | Mandatory |
|  | C-H Stretch, Alkyl | 3000 | 2855 | Variable | Mandatory |
| 33 | 89 | Aliphatic Sulphoxide | Aliphatic Sulfoxy-compound | Aliphatic Sulfur compound |  |
|  | Corresponding chemical | | | | |
|  | **Vibration** | **Start WN** | **End WN** | **Threshold** | **Priority** |
|  | S=O def | 520 | 400 | Weak | Medium |
|  | C-S stretch | 730 | 665 | Variable | Mandatory |
|  | S=O Stretch | 1050 | 1030 | Variable | Mandatory |
|  | C-H def | 1480 | 1420 | Variable | Mandatory |
|  | C-H stretch | 2990 | 2875 | Variable | Mandatory |
| 34 | 89 | Unsaturated Hydrocarbon, Cyclic, >C5 |  | Alkene |  |
|  | Corresponding chemical | | | | |
|  | **Vibration** | **Start WN** | **End WN** | **Threshold** | **Priority** |
|  | C-H bend, Alkene, OOP | 750 | 665 | Variable | High |
|  | C-H bend, Alkyl | 1470 | 1430 | Variable | High |
|  | C=C stretch, Alkene | 1650 | 1605 | Variable | Mandatory |
|  | C-H stretch, Alkyl | 3000 | 2900 | Variable | High |
|  | C-H Stretch, Alkene | 3090 | 3005 | Variable | High |
| 35 | 88 | Aliphatic acid Halide, Linear |  | Aliphatic acid Halide |  |
|  | Corresponding chemical | | | | |
|  | **Vibration** | **Start WN** | **End WN** | **Threshold** | **Priority** |
|  | X-C=O def | 540 | 460 | Variable | Medium |
|  | X-C=O, C-X stretch | 780 | 600 | Variable | Mandatory |
|  | C-C=O, C-C stretch | 960 | 910 | Variable | Very high |
|  | C-H bend, Alpha CH2 | 1210 | 1190 | Variable | Very high |
|  | C-H bend, CH2/CH3 | 1470 | 1445 | Variable | High |
|  | C=O stretch | 1825 | 1780 | Variable | Mandatory |
|  | C-H stretch, Alkyl | 3000 | 2855 | Variable | Medium |
| 36 | 87 | Phenyl substituent | Aryl group | Aromatic compound |  |
|  | Corresponding chemical | | | | |
|  | **Vibration** | **Start WN** | **End WN** | **Threshold** | **Priority** |
|  | C-H bend, OOP | 710 | 680 | Variable | Mandatory |
|  | C-H bend, OOP | 745 | 725 | Variable | Mandatory |
|  | C-C stretch, Ring | 1515 | 1485 | Variable | mandatory |
|  | C-H stretch, Aryl | 3090 | 3015 | Variable | Medium |
| 37 | 86 | Unsaturated Hydrocarbon, Vinylidene |  | Alkene |  |
|  | Corresponding chemical | | | | |
|  | **Vibration** | **Start WN** | **End WN** | **Threshold** | **Priority** |
|  | Skeletal | 560 | 530 | Variable | High |
|  | C-H bend, Alkene, OOP | 895 | 865 | Variable | Mandatory |
|  | C-H bend, Alkyl | 1480 | 1440 | Variable | High |
|  | C=C stretch, Alkene | 1665 | 1635 | Variable | Mandatory |
|  | C-H stretch, Alkyl | 3000 | 2900 | Variable | High |
|  | C-H stretch, Alkene | 3095 | 3050 | Variable | Mandatory |
| 38 | 85 | Alkene conjugated Ketone | Conjugated ketone | Aromatic or conjugated ketone |  |
|  | Corresponding chemical | | | | |
|  | **Vibration** | **Start WN** | **End WN** | **Threshold** | **Priority** |
|  | C=C-H, OOP | 985 | 930 | Variable | Mandatory |
|  | C-H bend | 1470 | 1430 | Variable | Mandatory |
|  | C=C stretch | 1640 | 1600 | Variable | Mandatory |
|  | C=O stretch | 1690 | 1660 | Strong | Mandatory |
| 39 | 84 | Carbonyl, Alpha Methylene | Substituent group | Side chain or substituent |  |
|  | Corresponding chemical | | | | |
|  | **Vibration** | **Start WN** | **End WN** | **Threshold** | **Priority** |
|  | C-H bend, CH3 | 1395 | 1350 | Variable | Mandatory |
|  | C-H bend, Alpha CH2 | 1425 | 1395 | Variable | Mandatory |
|  | C-H bend, CH2/CH3 | 1475 | 1450 | Variable | Mandatory |
|  | C=O stretch | 1850 | 1690 | Strong | Mandatory |
| 40 | 84 | Linear Bromo compound | Aliphatic Brominated compound | Aliphatic Halogen compound |  |
|  | Corresponding chemical | | | | |
|  | **Vibration** | **Start WN** | **End WN** | **Threshold** | **Priority** |
|  | C-Br stretch | 730 | 560 | Variable | Mandatory |
|  | C-H wag, Halogen substituted | 1245 | 1225 | Variable | Mandatory |
|  | C-H def, alpha CH | 1440 | 1385 | Variable | Mandatory |
|  | C-H bend, CH2/CH3 | 1465 | 1425 | Medium | Medium |
|  | C-H stretch, Alkyl | 3030 | 2900 | Variable | High |
| 41 | 84 | Unsaturated Hydrocarbon, Trans conjugated |  | Alkene |  |
|  | Corresponding chemical | | | | |
|  | **Vibration** | **Start WN** | **End WN** | **Threshold** | **Priority** |
|  | C-H bend, Alkene, OOP | 990 | 955 | Strong | High |
|  | C-H bend, Alkyl | 1470 | 1430 | Variable | Very high |
|  | C=C stretch, Alkene | 1660 | 1585 | Variable | Mandatory |
|  | C-H stretch, Alkyl | 3000 | 2900 | Variable | Very high |
|  | C-H stretch, Alkene | 3050 | 3010 | Variable | Mandatory |
| 42 | 83 | Silanol | Silanols | Aliphatic Silicon compound |  |
|  | Corresponding chemical | | | | |
|  | **Vibration** | **Start WN** | **End WN** | **Threshold** | **Priority** |
|  | Si-O stretch | 955 | 830 | Strong | Mandatory |
|  | Si-CH2 def | 1250 | 1175 | Variable | Very high |
|  | C-H stretch | 2990 | 2875 | Variable | Very high |
|  | O-H stretch | 3700 | 3200 | Variable | Mandatory |
| 43 | 83 | Unsaturated Hydrocarbons, Cis-Trans Alkene |  | Alkene |  |
|  | Corresponding chemical | | | | |
|  | **Vibration** | **Start WN** | **End WN** | **Threshold** | **Priority** |
|  | C-H bend, Alkene, OOP | 725 | 670 | Variable | Mandatory |
|  | C-H bend, Alkene, OOP | 975 | 950 | Variable | Mandatory |
|  | C-H bend, Alkyl | 1480 | 1440 | Variable | High |
|  | C=C stretch, Alkene | 1680 | 1620 | Variable | Mandatory |
|  | C-H stretch, Alkyl | 3000 | 2900 | Variable | High |
|  | C-H stretch, Alkene | 3035 | 3005 | Variable | Mandatory |
| 44 | 81 | Unsaturated Hydrocarbon, Cis Alkene |  | Alkene |  |
|  | Corresponding chemical | | | | |
|  | **Vibration** | **Start WN** | **End WN** | **Threshold** | **Priority** |
|  | Skeletal | 650 | 500 | Variable | High |
|  | C-H bend, Alkene, OOP | 725 | 670 | Variable | Mandatory |
|  | C-H bend, Alkyl | 1480 | 1440 | Variable | High |
|  | C=C stretch, Alkene | 1665 | 1620 | Variable | Mandatory |
|  | C-H stretch, Alkyl | 3000 | 2900 | Variable | High |
|  | C-H stretch, Alkene | 3035 | 3005 | Variable | Mandatory |
| 45 | 77 | Possibly Methyl Siloxane or Silane | Substituent group | Side chain or substituent |  |
|  | Corresponding chemical | | | | |
|  | **Vibration** | **Start WN** | **End WN** | **Threshold** | **Priority** |
|  | Si-C stretch | 860 | 820 | Strong | Mandatory |
|  | Si-CH3 (C-H bend) | 1260 | 1235 | Strong | Mandatory |
| 46 | 76 | Unsaturated Hydrocarbon, Trisubstituted |  | Alkene |  |
|  | Corresponding chemical | | | | |
|  | **Vibration** | **Start WN** | **End WN** | **Threshold** | **Priority** |
|  | Skeletal | 520 | 480 | Variable | High |
|  | Skeletal | 570 | 530 | Variable | High |
|  | C-H bend, Alkyl | 1480 | 1435 | Variable | Mandatory |
|  | C=C stretch, Alkene | 1695 | 1665 | Variable | Mandatory |
|  | C-H stretch, Alkyl | 3000 | 2900 | Variable | High |
|  | C-H stretch, Alkene | 3045 | 3005 | Variable | Mandatory |
| 47 | 72 | Aliphatic Tertiary |  | Aliphatic Amide |  |
|  | Corresponding chemical | | | | |
|  | **Vibration** | **Start WN** | **End WN** | **Threshold** | **Priority** |
|  | C-N stretch/C-C stretch | 1150 | 1050 | Variable | Mandatory |
|  | C-H bend | 1460 | 1410 | Variable | Mandatory |
|  | C=O stretch, Amido | 1690 | 1645 | Strong | Mandatory |
|  | C-H stretch, Alkyl | 3000 | 2855 | Variable | Medium |
| 48 | 72 | Ethoxy Silane | Silyl Ethers | Aliphatic Silicon compound |  |
|  | Corresponding chemical | | | | |
|  | **Vibration** | **Start WN** | **End WN** | **Threshold** | **Priority** |
|  | Si-O-C stretch, sym | 990 | 945 | Variable | Mandatory |
|  | Si-O-C stretch, asym | 1100 | 1070 | Variable | Mandatory |
|  | Et-O rock | 1200 | 1145 | Variable | Mandatory |
|  | C-H stretch | 2885 | 2855 | Variable | Very high |

**Table 2: Elemental analysis – correlation matrix**

| C VB | 1 |  |  |  |  |  |  |  |  |  |
| --- | --- | --- | --- | --- | --- | --- | --- | --- | --- | --- |
| **C NVB** | 0.99633 | 1 |  |  |  |  |  |  |  |  |
| **G1 O** | 0.99633 | 0.97897 | 1 |  |  |  |  |  |  |  |
| **G1 N** | 0.99633 | 0.86202 | 0.90416 | 1 |  |  |  |  |  |  |
| **G2 O** | 0.99633 | 0.97784 | 0.99753 | 0.91294 | 1 |  |  |  |  |  |
| **G2 N** | 0.99633 | 0.97915 | 0.99119 | 0.84499 | 0.98438 | 1 |  |  |  |  |
| **G3 O** | 0.99633 | 0.98633 | 0.97628 | 0.80606 | 0.96789 | 0.99295 | 1 |  |  |  |
| **G3 N** | 0.99633 | 0.92761 | 0.97708 | 0.85550 | 0.97767 | 0.97286 | 0.94317 | 1 |  |  |
| **G4 O** | 0.99633 | 0.96923 | 0.91010 | 0.72121 | 0.90352 | 0.93495 | 0.96897 | 0.84317 | 1 |  |
| **G4 N** | 0.99633 | 0.98389 | 0.93334 | 0.78339 | 0.93136 | 0.94759 | 0.97229 | 0.86647 | 0.99206 | 1 |
|  | **C VB** | **C NVB** | **G1 O** | **G1 N** | **G2 O** | **G2 N** | **G3 O** | **G3 N** | **G4 O** | **G4 N** |

Table 2: Elemental analysis - Correlation matrix of different developmental stages of galls. CVB: Control vascular bundle, C NVB: Control non-vascular bundle, G1 O: Gall 1^st^ stage outer region, G1 N: Gall 1^st^ stage inner region, G2 O: Gall 2^nd^ stage outer region, G2 N: Gall 2^nd^ stage inner region, G3 O: Gall 3^rd^ stage outer region, G3 N: Gall 3^rd^ stage inner region, G4 O: Gall 4^th^ stage outer region, G4 N: Gall 4^th^ stage inner region**.**

**Table 3: Elemental distribution pattern – Source-Sink status**

| Elements | Control weight %  Mean ± SD | | Weight %  T S of Gall –  1^st^  Mean ± SD | | Weight %  T S of Gall – 2^nd^  Mean ± SD | | Weight %  T S of Gall – 3^rd^  Mean ± SD | | Weight %  T S of Gall – 4^th^  Mean ± SD | | F test | |
| --- | --- | --- | --- | --- | --- | --- | --- | --- | --- | --- | --- | --- |
|  | **Non -VB** | **VB** | **In** | **Out** | **In** | **Out** | **In** | **Out** | **In** | **Out** | **F value** | **P Value** |
| B | 0.07 ± 0.01 | 0.43 ±0.02 | 0.06 ± 0.01 | 0.04 ± 0.01 | 0.04 ± 0.00 | 0.12 ± 0.01 | 0.07 ± 0.01 | 0.03 ± 0.00 | 0.12 ± 0.01 | 0.10 ± 0.01 | 257.589 | 0.000^**^ |
| C | 43.34 ± 1.64 | 41.86 ± 0.02 | 35.34 ± 0.06 | 41.16 ± 0.70 | 37.27 ± 0.02 | 49.23 ± 0.06 | 30.81 ± 0.01 | 43.86 ± 0.01 | 45.23 ± 0.03 | 51.96 ± 0.02 | 746.859 | 0.000^**^ |
| N | 0.46 ± 0.023 | 1.05 ± 0.04 | 1.02 ± 0.02 | 2.20 ± 0.16 | 1.25 ± 0.02 | 1.46 ± 0.02 | 1.85 ± 0.02 | 0.66 ± 0.02 | 1.18 ± 0.02 | 0.30 ± 0.01 | 643.769 | 0.000^**^ |
| O | 27.24 ± 0.03 | 26.04 ± 0.03 | 33.34 ± 0.04 | 36.03 ± 0.14 | 35.01 ± 0.02 | 40.08 ± 4.09 | 36.00 ± 0.01 | 36.15 ± 0.03 | 24.01 ± 0.01 | 28.24 ± 0.02 | 10.946 | 0.000^**^ |
| Fe | ND | ND | 0.17 ± 0.25 | 0.21 ± 0.02 | ND | 0.27 ± 0.02 | ND | ND | 0.52 ± 0.01 | 0.62 ± 0.02 | 47.048 | 0.000^**^ |
| Co | ND | ND | ND | 0.20 ± 0.02 | 0.01 ± 0.00 | ND | ND | ND | 0.52 ± 0.01 | ND | 2075.997 | 0.000^**^ |
| Cu | 0.01 ± 0.00 | ND | 0.06 ± 0.01 | 0.198 ± 0.01 | 0.17 ± 0.01 | 0.03 ± 0.01 | ND | ND | 0.63 ± 0.01 | ND | 2314.884 | 0.000^**^ |
| Zn | 0.04 ± 0.00 | ND | 0.01 ± 0.00 | 0.12 ± 0.02 | 0.04 ± 0.01 | 0.02 ± 0.01 | ND | ND | 0.75 ± 0.02 | ND | 2185.763 | 0.000^**^ |
| Na | 0.02 ± 0.00 | ND | 0.06 ± 0.01 | 0.30 ± 0.01 | 0.10 ± 0.01 | 0.01 ± 0.00 | 0.07 ± 0.01 | 0.04 ± 0.07 | 0.23 ± 0.02 | 0.01 ± 0.00 | 91.866 | 0.000^**^ |
| Mg | 0.22 ± 0.01 | ND | 0.06 ± 0.01 | 0.15 ± 0.01 | 0.12 ± 0.01 | 0.22 ± 0.14 | 0.13 ± 0.01 | 0.11 ± 0.01 | 0.64 ± 0.02 | 0.02 ± 0.00 | 83.853 | 0.000^**^ |
| Se | 0.23 ± 0.01 | ND | 0.09 ± 0.01 | 0.20 ± 0.01 | 0.045 ± 0.01 | 0.11 ± 0.05 | 0.03 ± 0.03 | 0.06 ± 0.01 | 0.10 ± 0.01 | 0.04 ± 0.00 | 56.663 | 0.000^**^ |
| Al | 0.15 ± 0.01 | ND | 0.16 ± 0.01 | 0.23 ± 0.02 | 0.13 ± 0.01 | 0.19 ± 0.01 | 0.2 ± 0.01 | 0.22 ± 0.09 | 0.66 ± 0.02 | 0.05 ± 0.00 | 154.519 | 0.000^**^ |
| Si | 0.43 ± 0.02 | 0.06 ± 0.01 | 0.1 ± 0.01 | 0.28 ± 0.01 | 0.76 ± 0.01 | 0.56 ± 0.02 | 0.21± 0.01 | 0.43 ± 0.18 | 0.96 ± 0.02 | 0.23 ± 0.01 | 132.763 | 0.000^**^ |
| P | 7.53 ± 0.03 | 8.55 ± 0.03 | 11.12 ± 0.03 | 12.25 ± 0.03 | 9.24 ± 0.03 | 17.83 ± 0.03 | 18.10 ± 0.02 | 7.12 ± 0.01 | 4.85 ± 0.03 | 2.73 ± 0.02 | 80366.200 | 0.000^**^ |
| Hg | 0.50 ± 0.01 | 0.67 ± 0.02 | 0.41 ± 0.01 | 1.32 ± 0.02 | 0.66 ± 0.01 | 1.62 ± 0.02 | 0.17 ± 0.01 | 1.60 ± 0.01 | 2.98 ± 0.02 | 2.56 ± 0.02 | 5144.089 | 0.000^**^ |
| Mo | 0.54 ± 0.03 | 0.11 ± 0.01 | 0.01 ± 0.00 | 0.09 ± 0.15 | 0.01 ± 0.00 | 0.14 ± 0.01 | ND | 0.11 ± 0.01 | 0.21 ± 0.01 | 0.02 ± 0.01 | 60.996 | 0.000^**^ |
| Cl | 0.35 ± 0.02 | 0.08 ± 0.01 | 0.45 ± 0.01 | 0.20 ± 0.01 | 0.25 ± 0.01 | 0.27 ± 0.02 | 0.31 ± 0.01 | 0.25 ± 0.01 | 1.43 ± 0.03 | 0.26 ± 0.01 | 2247.311 | 0.000^**^ |
| K | 15.94 ± 0.02 | 19.42 ± 0.03 | 42.75 ± 0.03 | 23.15 ± 0.10 | 15.32 ± 0.02 | 28.08 ± 0.08 | 18.23 ± 0.02 | 12.45 ± 0.02 | 10.24 ± 0.01 | 5.64 ± 0.23 | 86198.638 | 0.000^**^ |
| Ca | 0.34 ± 0.02 | 0.04 ± 0.01 | 0.35 ± 0.02 | 0.26 ± 0.01 | 0.25 ± 0.01 | 0.21 ± 0.01 | 0.16 ± 0.01 | 0.12 ± 0.01 | 1.49 ± 0.01 | 0.36 ± 0.02 | 3359.643 | 0.000^**^ |
| I | 0.67 ± 0.01 | 0.35 ± 0.01 | 1.16 ± 0.021 | 0.54 ± 0.03 | 1.74 ± 0.01 | 1.43 ± 0.01 | 1.03 ± 0.02 | 0.98 ± 0.01 | 4.21 ± 0.02 | 0.75 ± 0.02 | 729.257 | 0.000^**^ |
| Mn | 1.66 ± 0.02 | 1.23 ± 0.02 | 1.70 ± 1.70 | 1.21 ± 0.01 | 1.75 ± 0.02 | 1.84 ± 0.03 | 1.48 ± 0.02 | 1.42 ± 0.02 | 3.43 ± 0.02 | 1.34± 0.03 | 42.18.510 | 0.000^**^ |

Table 4: NB: Non-Vascular bundle, VB: Vascular Bundle, In: Inner region, Out: Outer region and ND: Not detected. (** significance level at 0.001).

**Fig. 1: Complex gall structure**


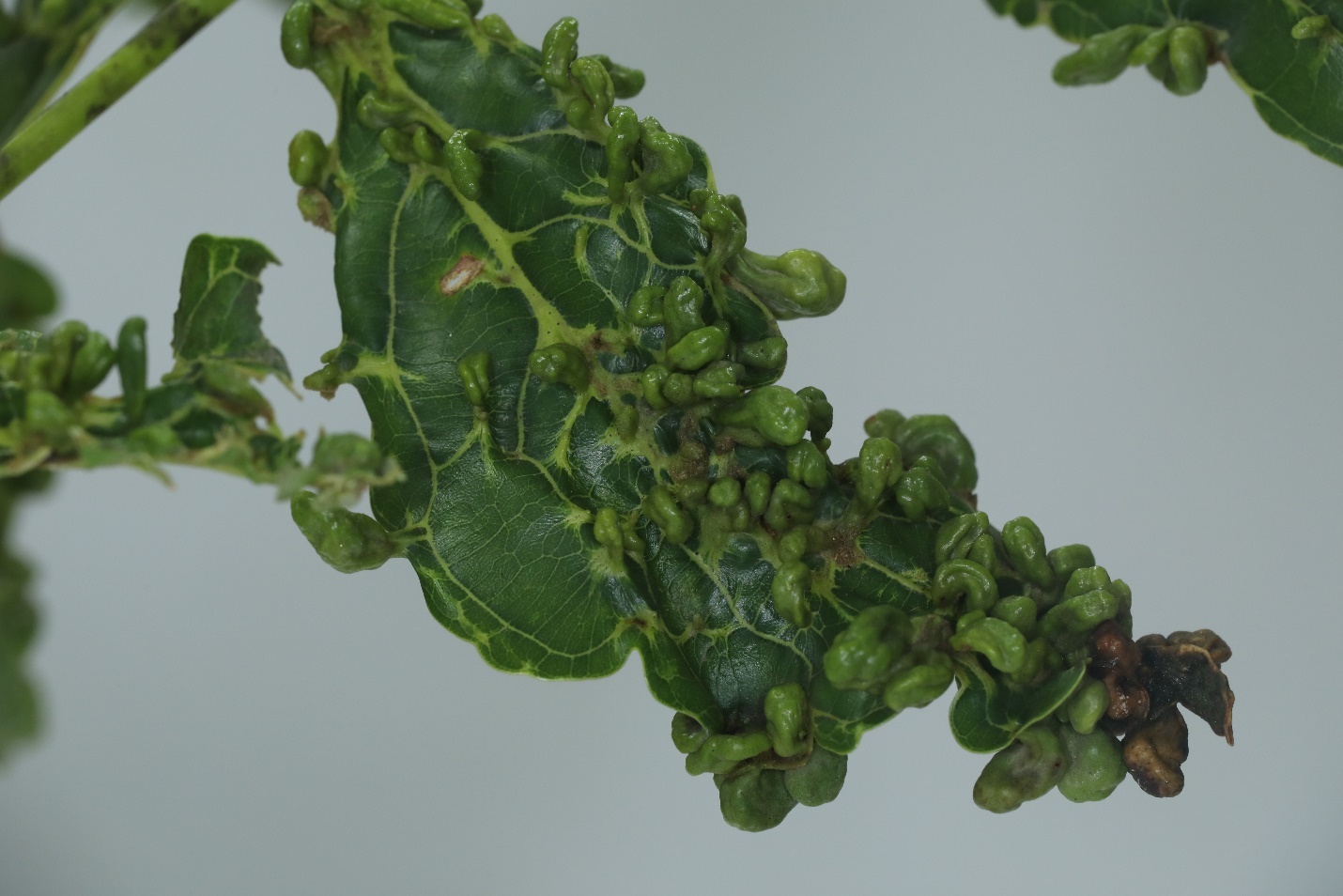


Table. 4: ATR-FTIR Correlation matrix of different developmental stages of gall

|  | Control | Gall 1^st^ stage | Gall 2^nd^ stage | Gall 3^rd^ stage | Gall 4^th^ stage |
| --- | --- | --- | --- | --- | --- |
| Control | 1 | 0.75002 | 0.76637 | 0.78806 | 0.59230 |
| 1^st^ stage Gall | 0.75002 | 1 | 0.99133 | 0.98655 | 0.95828 |
| 2^nd^ stage Gall | 0.76637 | 0.99133 | 1 | 0.99372 | 0.92990 |
| 3^rd^ stage Gall | 0.78806 | 0.98655 | 0.99372 | 1 | 0.92000 |
| 4^th^ stage Gall | 0.59230 | 0.95828 | 0.92990 | 0.92000 | 1 |
